# Axially-chiral boramidine for detailed (chir)optical studies†

**DOI:** 10.1039/d4sc00870g

**Published:** 2024-04-02

**Authors:** Nidal Saleh, Estefanía Sucre-Rosales, Francesco Zinna, Céline Besnard, Eric Vauthey, Jérôme Lacour

**Affiliations:** a Department of Organic Chemistry, University of Geneva Quai Ernest Ansermet 30 1211 Geneva 4 Switzerland nidal.saleh@unige.ch jerome.lacour@unige.ch; b Department of Physical Chemistry, University of Geneva Quai Ernest Ansermet 30 1211 Geneva 4 Switzerland eric.vauthey@unige.ch; c Dipartimento di Chimica e Chimica Industriale, University of Pisa Via G. Moruzzi 13 56124 Pisa Italy; d Laboratory of Crystallography, University of Geneva Quai Ernest Ansermet 24 1211 Geneva 4 Switzerland

## Abstract

The inclusion of boron atoms into chiral π-conjugated systems is an effective strategy to unlock unique chiroptical properties. Herein, the preparation and characterization of a configurationally stable axially-chiral boramidine are reported, showcasing absorption in the UV domain, deep-blue fluorescence (*Φ* up to 94%), and *ca.* |10^−3^| *g*_abs_ and *g*_lum_ values. Detailed photophysical studies and quantum-chemical calculations clearly elucidate the deactivation pathways of the emissive state to triplet excited states, involving increased spin–orbit coupling between the lowest singlet excited state and an upper triplet state.

## Abstract 2

An axially-chiral boramidine displays UV-absorption, deep-blue fluorescence (*Φ* up to 94%), and |10^−3^| *g*_abs_ and *g*_lum_. Deactivation pathways of the emissive state to triplet excited states, involving increased spin–orbit coupling, are identified.

## Graphical abstract

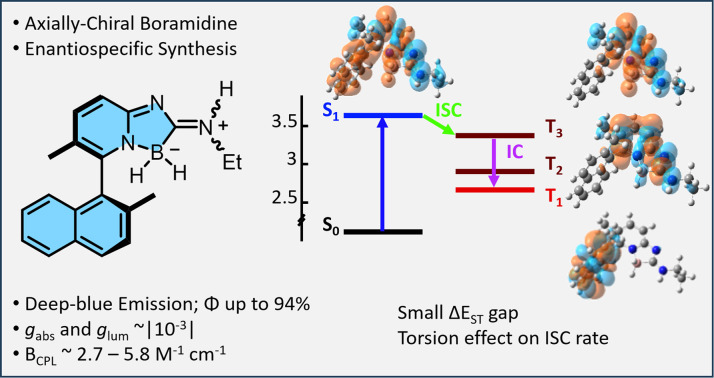

## Introduction

**Fig. 1:**
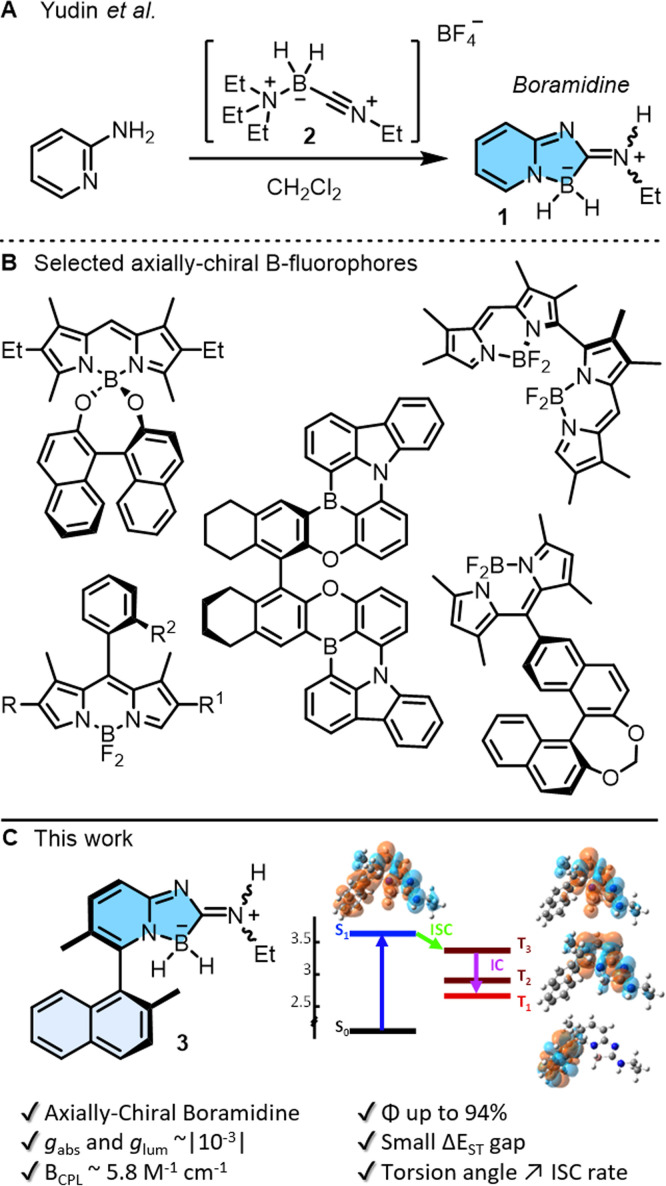
(A) Synthesis of boramidine 1 by reaction of α-amino pyridine with *N*-alkylnitriliumborane 2.^6^ (B) Boron-containing axially chiral fluorophores. (C) Enantiospecific synthesis of axially-chiral 3 and subsequent (chir)optical properties; (−)-(*M* or *R*)-enantiomer shown arbitrarily.

Small organic molecules exhibiting circularly polarized luminescence are attracting well deserved attention. These chiral chromophores, often abbreviated as SOM-CPL, are applied in cutting-edge research, ranging from 3D displays and optical sensors, to optical information storage or encryption.^1^ To address (chir)optical properties, main group elements can be incorporated into the π-conjugated systems. This strategy, often allied with efficient preparation routes, unlocks unique optoelectronic properties unattainable with traditional carbon- or metal-based materials.^2^ In this context, boron atoms are remarkably useful in either three or four-coordinated geometries^3^ and chiral B-containing derivatives can be readily generated with stereogenic elements of central, axial, planar or helical chirality.^1,4,5^ Recently, a new class of achiral boramidine chromophores 1 were prepared by simple treatment of α-amino pyridine with *N*-alkylnitriliumborane 2 in one step (Fig. 1A).^6^ Initial results by Yudin and coworkers indicated remarkable stability and high fluorescence quantum yields (up to 68%), making compounds 1 excellent candidates for SOM-CPL if transformed into chiral enantiopure moieties. Of all the previously mentioned chiral emitters,^1,4,5^ axially chiral derivatives are remarkable by the simplicity of their design, the ease of their synthesis, and the tuning of CPL emission by the control of the biaryl twist (dihedral angle) (Fig. 1B).^5^ Herein, the preparation, resolution and properties of axially-chiral boramidine 3 are reported (Fig. 1C). It displays absorption in the UV domain, narrow deep-blue fluorescence, large Stokes shifts, strong absorption and luminescence dissymmetric factors (*g*_abs_ and *g*_lum_ ∼10^−3^) in electronic circular dichroism (ECD) and CPL. Of note, and contrary to the initial report,^6^ detailed time-resolved photophysical studies and quantum-chemical calculations unravel new deactivation pathways of the emissive state of 1 and 3, *via* triplet states in particular. The results further reveal that the introduction of axial chirality to the boramidine enhances spin–orbit coupling and facilitates intersystem crossing from the emissive state to the triplet manifold; a constraint not yet identified but of key importance in the design of biaryl-type fluorophores.

## Results and discussion

### Synthesis

**Scheme 1:**
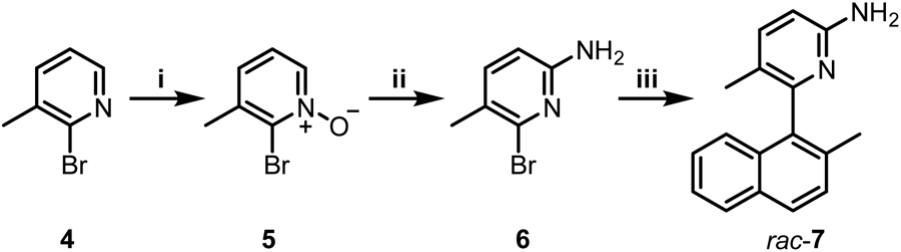
(i) *m*-CPBA, CH_2_Cl_2_, 0 to 25 °C, 16 h, 84%; (ii) (a) trifluoroacetic anhydride, pyridine, CH_2_Cl_2_, 0 to 25 °C, 6 h; (b) ethanolamine, ACN, 16 h, 74%; (iii) (2-methylnaphthalen-1-yl)boronic acid, Pd(PPh_3_)_4_, Na_2_CO_3_, toluene : EtOH : H_2_O, 100 °C, 20 h, 97%.

Boramidines 1 have been first and only reported in 2020 as achiral materials.^6^ To create an asymmetric variant, the construction of axially-chiral 3 was considered knowing that metal-catalyzed cross-coupling reactions would offer efficient and direct synthetic routes to the core structure. 2-Amino-5-bromo-3-methylpyridine precursor 6 was prepared in two steps: firstly, the oxidation of commercially available 4 with *m*-CPBA to afford pyridine oxide 5 in 84% yield followed by a Zincke aminolysis to reach 6 in 74%; and secondly a Pd-catalyzed Suzuki–Miyaura cross-coupling reaction of 6 with (2-methylnaphthalen-1-yl)boronic acid which finally yielded *rac*-7 in essentially quantitative yield (97%) (Scheme 1).

**Scheme 2:**
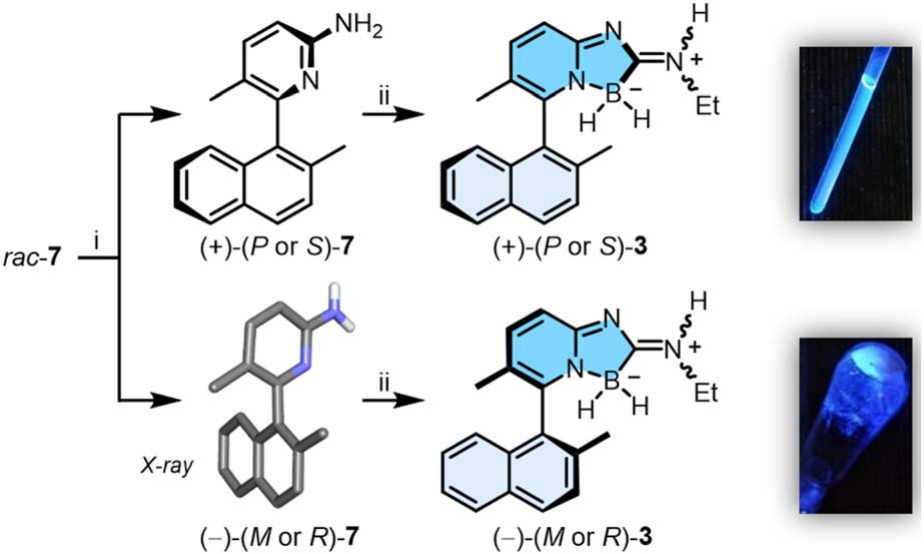
Synthesis of (+) and (−)-3: (i) CSP-HPLC resolution (CHIRALPAK® IC, ^i^PrOH : hexane (+0.1% Et_2_NH), 5 : 95) and X-ray structure of (−)-(*M* or *R*)-7 (most H-atoms omitted); (ii) 2, CH_2_Cl_2_, 16 h, r.t., 68–70% yields. Right: CH_2_Cl_2_ solution and solid-state fluorescence (*λ*_exc_ = 366 nm).

Resolution by chiral stationary phase (CSP) HPLC afforded rapidly and efficiently the single enantiomers (+) and (−)-7, which were obtained as first and second eluted fractions in excellent yields (>45%) and enantiomeric purity (>99% ee, Fig. S22 and S23†).^7^ Furthermore, samples of (−)-7 crystallized in chloroform, and their X-ray structural analysis revealed (*R*) or (*M*) absolute configurations for the chiral axis (Table S4 and Fig. S24†). With these (−)-(*M* or *R*) and (+)-(*P* or *S*) enantiomers of 7 in hand, by reaction with a slight excess of 2 (1.5 equiv.), (+)- and (−)-3 were prepared in 70% and 68% yields, respectively (Scheme 2). Of note, a 4 : 1 *E* : *Z* ratio is noticed for the exocyclic iminium appendage with either racemic and enantiopure samples, according to ^1^H and ^13^C NMR spectroscopy. In our hands, this ratio remained the same in various solvents and at different temperatures indicating that, for compound 3, an equilibrium between the *E* : *Z* configurations is unlikely.^8^ The geometrical isomers cannot be separated from one another and further studies have been performed with this mixture composition. Surprisingly, analytical CSP-HPLC conditions could not be found for enantiomers of 3 and the enantiospecificity (e.s. > 90%) of reaction 7 → 3 could only checked by ^1^H NMR spectroscopy using [Bu_4_N][*Δ*-BINPHAT] (Fig. S25 and S26†).^9,10^ Finally, care was taken to verify the configurational stability of compounds 3 at higher temperatures (*vide infra*). A series of ECD experiments were conducted with gradual temperature increase from 20 to 100 °C. The ECD spectra of (+)-3 (Fig. S10†), measured for periods of 30 minutes at the different increasing temperatures, remained unchanged. Compounds of type 3 are thus configurationally stable over a large range of temperatures and present hence a racemization barrier higher than 28 kcal mol^−1^.^11^ In view of the enantiospecificity and configurational stability of 3, absolute (*P* or *S*) and (*M* or *R*) configurations can be assigned with confidence for the (+)- and (−) enantiomers respectively.

### Photophysical and chiroptical measurements

**Fig. 2:**
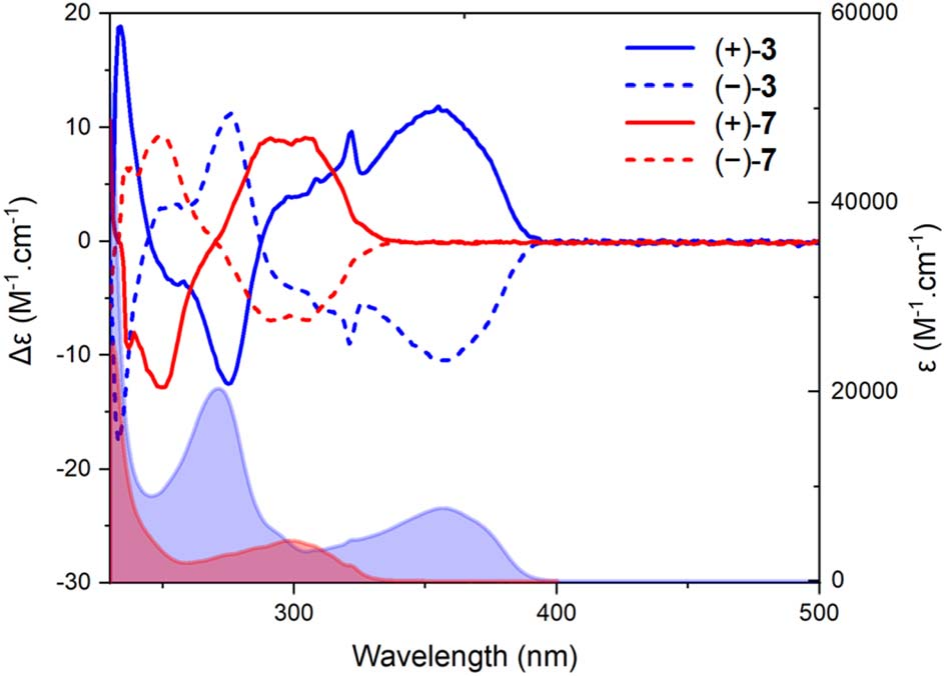
Electronic absorption and ECD spectra of 7 (red) and 3 (blue) in CH_2_Cl_2_ (2 × 10^−5^ M). Continuous (+)-(*P*) and dotted (−)-(*M*) lines.

**Fig. 3:**
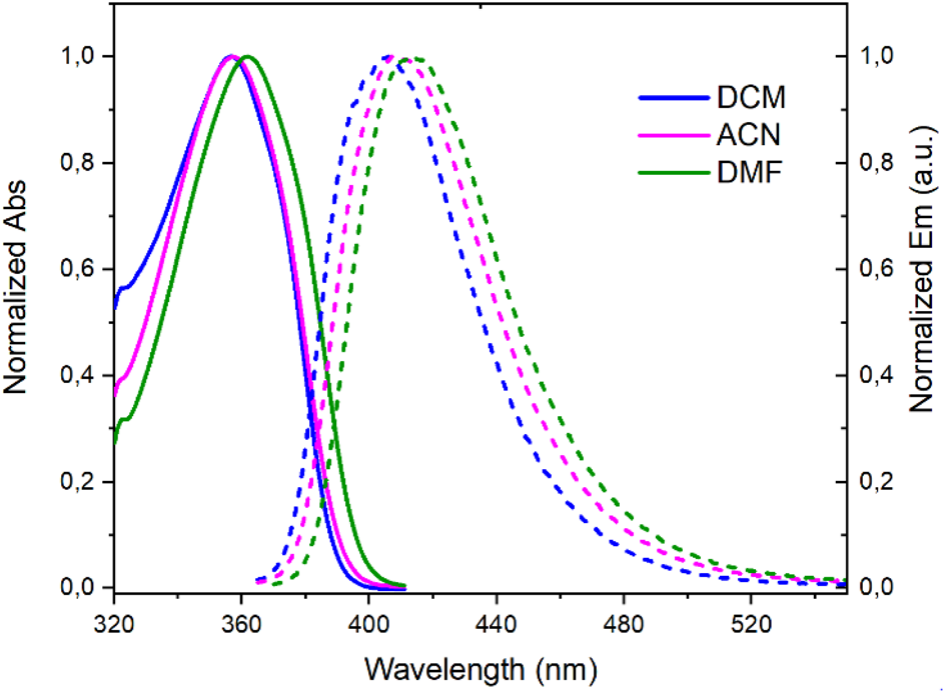
Normalized absorption (solid lines) and emission (dashed lines) spectra of 3 in CH_2_Cl_2_ (DCM), CH_3_CN (ACN), and DMF.

A deep-blue fluorescence for *rac*, (+)- and (−)-3 was apparent both in solution and solid states (Scheme 2). The photophysical properties were first investigated by steady-state absorption and emission measurements in solution. In DCM (CH_2_Cl_2_), the absorption spectrum of 3 was compared to that of 7. As presented in Fig. 2, a new absorption band appears in the 300–400 nm domain, with a *λ*_max_ at 357 nm and an extinction coefficient (*ε*) of 7720 M^−1^ cm^−1^. The absorption spectra of the chiral 3 and achiral 1 boramidines, used as a reference, present similar bands with the lower energy transition of 3 being slightly red-shifted (+7 nm, −560 cm^−1^) when compared to 1 (Fig. S1†).^12^ The intense absorption band centered at 225 nm in 3 corresponds to the contribution of the naphthalene moiety.^13^ A weak solvatochromism is noticed with blue and red shifts in MeOH (−7 nm, +560 cm^−1^) and DMF (+5 nm, −387 cm^−1^), respectively (Table 1 and Fig. 3, S2–S7†).

**Table 1:** Photophysical data of 3^a^

Solvent	*λ*_max_ (nm)	*ε* b (M^−1^ cm^−1^)	*λ*_em_ (nm)	Stokes shift (cm^−1^)	*Φ* c (*Φ*_N_2__) %	*g*_abs_ b (10^−3^)	*g*_lum_ d (10^−3^)	*B*_CPL_ (M^−1^ cm^−1^)
MeOH	350	9135	406	3940	66 (87)	+1.1/−1	+1/−1.3	5.1^e^
DCM	357	7720	406	3381	59	+1.5/−1.4	+1/−1.2	2.7
ACN	357	8820	407	3441	64 (89)	+1.2/−1.2	+1/−1	3.9^e^
Toluene	360	7650	404	3025	74	+1.1/−1	+1/−1.3	3.6
DMF	362	10 360	414	3470	79 (94)	+1/−1.1	+1.1/−1.2	5.8^e^

a Concentrations 2 × 10^−5^ M.

b Calculated for the most red-shifted band 350–362 nm.

c Diphenylanthracene (*λ*_exc_ = 373 nm, *Φ* = 97% in cyclohexane).

d *λ*_exc_ = 365 nm.

e N_2_-saturated solution.

The emission spectra spanned the 370–520 nm range, peaking at approximately 404–407 nm in toluene, MeOH, DCM, and ACN (CH_3_CN), and 414 nm in DMF (Table 1 and Fig. S8†). These spectra displayed a single narrow band with a full width at half maximum (FWHM) around 3000 cm^−1^ (Table S1†), accompanied by Stokes shifts within the 3400–3900 cm^−1^ range.

These features are consistent with high fluorescence quantum yields (*Φ*) of 64% and 79% in ACN and DMF, respectively. However, the emission intensity was dependent on the presence of dioxygen in solution; *Φ* increasing significantly to 89% and 94% under N_2_ saturation (*Φ*_N_2__, Table 1 and Fig. S9†).

**Fig. 4:**
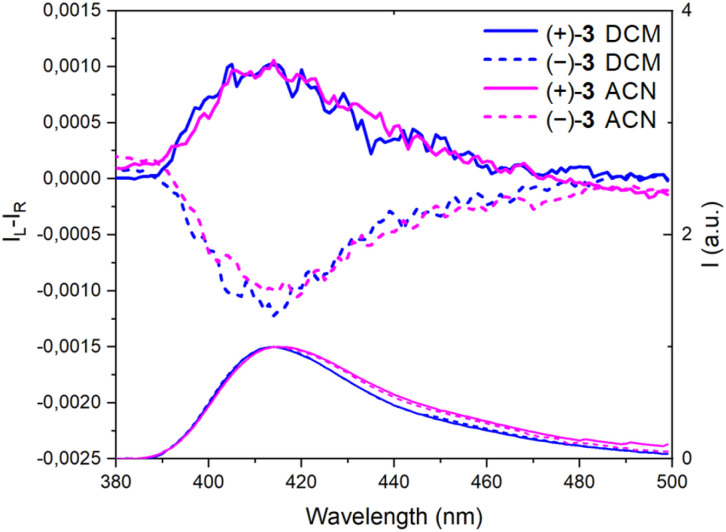
CPL (Top) and emission spectra (bottom) of (+)-(*P*) and (−)-(*M*)-3, continuous and dotted lines respectively, in CH_2_Cl_2_ (DCM) and CH_3_CN (ACN).

Then, the corresponding chiroptical spectroscopies were investigated using (+)- and (−)-3 (4 : 1 *E* : *Z* mixture). The ECD spectra were recorded for each enantiomer in the UV domain and, as expected, mirror-image Cotton effects were obtained (Fig. 2). A comparison with precursors (+)- and (−)-7 demonstrates relatively large differences in spectral shape with (same sign) lower energy signals around 300 nm for 7 and at 357 nm for 3, with |Δ*ε*| *ca.* 9 and 11 M^−1^ cm^−1^, respectively. However, the ECD measurements of 3 in MeOH, ACN, toluene and DMF did not reveal any significant change (Fig. S4–S7†). Overall, |*g*_abs_| values between 1.0 and 1.5 × 10^−3^ were obtained in various solvents (Table 1). All spectra present a feature around 320 nm which is possibly the result of a partial cancellation of two close-in-energy bands with opposite rotatory strength (Fig. 2 and S4–S7).† Interestingly, in CPL, the dextro and levoratory enantiomers of 3 displayed significant positive and negative signals in the visible domain (Fig. 4 and S8†), with luminescence dissymmetry factors (|*g*_lum_|) of 10^−3^. Of note, changing solvents or performing measurements in either aerobic or N_2_-saturated conditions did not modify the *g*_lum_ values. Finally, comparable *g*_abs_ and *g*_lum_ were hence found in the case of (+)- and (−)-3. This similarity indicates that only minor differences are present between the ground and excited state geometries, and it is consistent with previous correlations.^14^ The calculated CPL brightness (*B*_CPL_ = ½ *εΦ*|*g*_lum_|) magnitude is of approximately 2.7–5.8 M^−1^ cm^−1^ depending on the solvent, and it is similar to values reported for helicenes.^4*n*^

To better understand the role of O_2_ on the fluorescence and look for a possible involvement of triplet excited states in the deactivation pathways, transient absorption (TA) spectroscopy measurements combined with quantum chemical calculations at the TD-DFT level were carried out. To this end, 3 was compared with the unsubstituted 1.

**Fig. 5:**
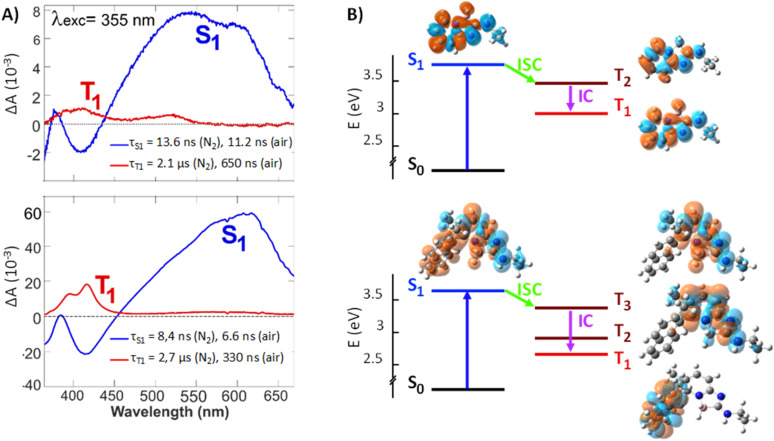
(A) Evolution-associated difference spectra resulting from a global analysis of the transient absorption data measured with 1 (top) and 3 (bottom) in ACN upon 355 nm excitation assuming two successive exponential steps (A → B →). (B) Mechanism for population of the lowest triplet state, together with the charge density difference isosurfaces (plotted at |Δ*ρ* = 0.0004|, orange and cyan colors represent, respectively, the increase and decrease of electronic density upon transition from the ground state).

The evolution-associated difference absorption spectra (EADS) obtained from a global analysis of the TA data in ACN assuming two successive exponential steps (A → B →) are depicted in Fig. 5, whereas the TA spectra are shown in Fig. S12 and S13.†

The spectra measured within the first ten ns are similar for both compounds and exhibit two negative features, which can be assigned to the ground state bleach (<370 nm) and the stimulated S_1_ → S_0_ emission (SE, around 420 nm), and a broad positive band above 500 nm. This band can be attributed to a S_*n*>1_ ← S_1_ absorption, as it decays in parallel to the SE with a similar time constant as the fluorescence lifetime measured by time-correlated single photon counting (TCSPC, Table S2 and Fig. S11†). Concurrently to the decay of the spectral features of the S_1_ state, new positive bands appear, the most intense being around 420 nm. These later spectra can be attributed to the lowest triplet state, T_1_, of both compounds as their decay accelerates in the presence of oxygen. The triplet spectrum of 3 with its vibronic structure differs significantly from that of 1 and resembles closely that of naphthalene.^15^

Gas-phase quantum-chemical calculations were performed to better understand the transition from the boramidine-centered S_1_ state to the naphthalene-centered T_1_ state of 3. The optimized equilibrium geometries of 1 in the ground (S_0_) and excited (S_1_) states point to minor structural changes upon photoexcitation. This is consistent with the chiroptical data shown above. For 3, the main difference concerns the dihedral angle between the aromatic planes, which decreases from 86° to 68° upon going to the S_1_ state (Fig. S14†). The energy-level diagrams obtained from the TD-DFT calculations are presented in Fig. 5. For both molecules, the calculated S_1_ energy is larger by 0.4 eV from that measured in ACN. These calculations predict the presence of two or three triplet states below the S_1_ state of 1 and 3, respectively. The charge density difference (CDD) isosurfaces, which reflect the changes of electronic density upon transition from the ground to a given state, confirm that the S_1_ and T_1_ states of 3 are mostly localized on the boramidine and naphthalene sub-units, respectively, as inferred from the TA spectra. Furthermore, the T_2_ and T_3_ states of 3 are mostly localized on the boramidine and resemble the T_1_ and T_2_ states of 1. For boramidine 1, calculations predict the spin–orbit coupling (SOC) constant for the S_1_→ T_2_ transition to be four times as large as that for the S_1_ → T_1_ transition (Fig. S17†). Given that the ISC rate constant scales with the square of the SOC constant and that the small S_1_ – T_2_ gap favors a large Franck–Condon factor, the transition to the triplet manifold should mostly occur *via* S_1_ → T_2_ ISC. The subsequent internal conversion to the T_1_ state can be expected to occur within a few ps or less.^16^ Therefore, the T_2_ state is too short lived to be observable in a TA experiment. In the case of 3, SOC calculations predict S_1_ → T_3_ ISC to be the dominant pathway to the triplet manifold (Fig. S19†). This implies that, upon ISC, the excitation remains mostly localized on the boramidine sub-unit before migrating to the naphthalene group upon internal conversion to the T_1_ state. Direct S_1_ → T_1_ ISC can be expected to be relatively inefficient, because of the large S_1_ – T_1_ gap and the relatively weaker SOC. The shorter fluorescence lifetime of 3 relatively to 1 can be accounted by a faster ISC, which itself can be explained by a S_1_ – T_3_ SOC that is larger than the corresponding S_1_ – T_2_ SOC of boramidine 1. This difference most probably arises from a partial delocalization of the S_1_ excitation on the naphthalene substituent, conferring some twisted character to the excited state.^17^ According to quantum-chemical calculations (Table S3 and Fig. S20†), the S_1_ – T_3_ SOC of 3 depends critically on the dihedral angle between the aromatic planes of the boramidine and naphthalene subunits and decreases upon decreasing this angle below its equilibrium value (Fig. S21†). To conclude this section, these results reveal that the helicity introduced to confer an axial chirality to 3 has a detrimental effect on the fluorescence quantum yield as it favors conversion to the triplet state; *Φ*_N_2__ values remaining still above 59% for 3.

## Conclusions

In this study, through the introduction of atropisomeric conformations, enantiopure boramidines were prepared as configurationally-stable fluorophores. Absorption in the UV domain and deep-blue fluorescence (*Φ* up to 94% in N_2_ saturated solutions) are noted and corresponding |*g*_abs_| and |*g*_lum_| values around 10^−3^ are further obtained in ECD and CPL for the excitation and emission dissymmetry factors. Detailed photophysical studies and quantum-chemical calculations helped elucidate deactivation pathways of the emissive state to triplet excited states of achiral and chiral boramidines. Of note, with chiral derivative 3, the necessary torsion (80–90°) between boramidine and naphthyl subunits favors spin–orbit coupling and accelerates intersystem crossing from the emissive state to the triplet manifold. In future studies, finding the right torsional balance to induce sufficient axial chirality without being detrimental to the fluorescence quantum yield should be considered in the design of biaryl-type fluorophores.

## Data availability

The data that support the findings of this study are openly available in https://yareta.unige.ch at https://doi.org/10.26037/yareta:xthmovjv45ccxghfhkdo7oq2ai. It will be preserved for 10 years.

## Author contributions

N. S. and J. L. conceived the original idea. N. S. designed the research approach, synthesized compounds, performed experiments and conducted data analysis. E. S.-R. and E. V. performed transient absorption and quantum-chemical calculations. F. Z. and C. B. completed CPL and X-ray structural analyses, respectively. J. L. supervised the project. N. S. and J. L. wrote the final manuscript with input and approval from all authors.

## Conflicts of interest

There are no conflicts to declare.

## Supplementary Material

SC-015-D4SC00870G-s001

SC-015-D4SC00870G-s002
