# Supplementary material for: Axially-chiral boramidine for detailed (chir)optical studies
Source: Chem Sci. 2024 Apr 2;15(17):6530–5. doi: 10.1039/d4sc00870g (PMC11062121; doi:10.1039/d4sc00870g)
Supplement: SC-015-D4SC00870G-s001 [file SC-015-D4SC00870G-s001.pdf]

Electronic Supplementary Material (ESI) for Chemical Science.
This journal is © The Royal Society of Chemistry 2024

Axially-Chiral Boramidine for Detailed (Chir)Optical Properties

Nidal Saleh,*[a] Estefanía Sucre-Rosales,[b] Francesco Zinna,[c] Céline Besnard,[d] Eric Vauthey,[b]* and Jérôme Lacour*[a]

Contents

I. General remarks S2
II. Synthesis S6
III. (Chir)Optical Measurements S9
IV. Time-Resolved Fluorescence S15
V. Transient Absorption Spectra S16
VI. Quantum-Chemical Calculations S17
VII. CSP-HPLC Resolution S24
VIII. Crystallographic Data S26
IX. NMR Spectra S28
X. References S34

I. General remarks

Reagents and solvents

Unless otherwise stated, reagents were purchased from commercial sources and used without further purification. All reactions involving air sensitive compounds were carried out under N_2 via an inert gas/vacuum double manifold line and standard Schlenk techniques using dry solvents.

Chromatography

Flash column chromatography was performed with silica gel 40-63 µm.

Nuclear magnetic resonance (NMR)

NMR spectra were recorded on Bruker Avance III 500 MHz, Bruker Avance III HD-NanoBay 400 MHz and Bruker Avance III HD-NanoBay 300 MHz spectrometers at room temperature. ^{13}C-NMR: chemicals shifts were given in ppm relative to Me_4Si with solvent resonances used as internal standards (CD_2Cl_2 δ= 5.32, 54.00 ppm). Data were reported as follows: chemical shift (δ) in ppm on the δ scale, multiplicity (s= singulet, d= doublet, t= triplet, dd= doublet of doublets, td= triplet of doublets, m= multiplet), coupling constant (Hz) and integration.

Infrared Spectroscopy

IR spectra were recorded on a Perkin-Elmer 1650 FT-IR spectrometer using a diamond ATR Golden Gate sampling, and are reported in wave numbers (cm^{-1}).

Melting points (M.p.)

M.p. were measured in open capillary tubes and were uncorrected.

High-resolution Mass Spectrometry (HRMS)

Electrospray mass spectra were obtained on a Xevo-G2-TOF HRMS by the Department of Mass Spectroscopy of the University of Geneva.

Chiral Stationary Phase (CSP) HPLC resolutions

Semi-preparative Chiral stationary phase HPLC resolutions were performed on an Agilent LC 1100 instrument using CHIRALPAK® IC column.

(Chir)Optical Measurements All (chir)optical measurements were performed in 1cm optical quartz cells.

Steady-State Absorption

Spectra were recorded on a JASCO V-650 spectrophotometer at 20°C with parameters as follows: scan speed – 100 nm/min, bandwidth – 1 nm, and data interval – 1 nm. All samples were measured in 1 cm optical path quartz cells. All solvents were of spectroscopic or HPLC grade and were used as received.at precise concentrations ca. 2 10^{-5} M.

Electronic Circular dichroism (ECD)
Spectra were recorded on either JASCO J-815 or JASCO J-1500 spectropolarimeters at 20°C with parameters as follows: scan speed – 100 nm/min, bandwidth – 1 nm, data interval – 1 nm, and integration time – 0.1 sec. Measurement were performed in analytical grade solvents in 1 cm optical path quartz cells at precise concentrations ca. 2 10^{-5} M. All the spectra are the average of 10 accumulations each.

Steady-State Emission
Spectra were measured using a FluoroMax+ spectrofluorometric from Horiba scientific with parameters as follows: Entrance and Exit slit – 1 nm bandpass, integration time – 0.1 sec. All fluorescence spectra were corrected for the wavelength-dependent sensitivity of the detection. Fluorescence quantum yields Φ were measured in diluted solutions with an optical density lower than 0.1 relative to 9,10-diphenylanthracene (Φ_r = 0.97 in cyclohexane) using the following equation:

$$\Phi = \Phi_r \frac{I\, A_r\, n^2}{I_r\, A\, n_r^2}$$

where *A* is the absorbance at the excitation wavelength (λ), *n* the refractive index and *I* the integrated emission intensity; "*r*" stands for reference. Excitations of reference and sample compounds were performed at the same wavelength 373 nm.

Circularly Polarized Luminescence (CPL)
Measurements were performed using a homemade spectrofluoropolarimeter[1] under 365 nm irradiation from commercial LED-sources with a 90° geometry between excitation and detection. The spectra were run in DCM and ACN in ~$2\cdot10^{-5}$ M solutions using the following parameters: scan-speed – 2 nm/s, integration time – 2 or 4 s, photomultiplier tube driving voltage – from 400 to 650 V, emission bandwith ca. 10 nm. All the spectra are the average of 4 accumulations each.

Specific Optical Rotation
$[\alpha]_{20}^{D}$ were measured using JASCO P-1030 polarimeter. Measurements were performed in analytical grade CH_2Cl_2 solvent at precise concentrations in a Faraday cell Pb glass, temperature 20°C, and sodium lamp.

Time-Correlated Single Photon Counting (TCSPC) Experiments
The samples were bubbled with nitrogen or air at least 15 minutes before the measurement, and sealed. The TCSPC experiments were performed at the magic angle, using a 375 nm laser diode (PicoQuant LDH-P-C-375) as the excitation source, and recording the emission at 400 nm. Such emission was collected in an optical fiber, filtered using a spectrograph (Horiba Triax 190), and finally detected using a photomultiplier tube (PicoQuant PMA 192C). The instrument response function (IRF) had a full width at half maximum of about 200 ps. The fluorescence lifetimes were determined from iterative reconvolution of a monoexponential function with the IRF.

Transient Absorption (TA) Spectroscopy

Before experiments, the samples were bubbled for at least 15 minutes with nitrogen or air. All TA experiments were performed at the magic angle, keeping the samples under nitrogen or airflow during the entire duration of the measurement.

The ps-μs pump-probe setup is described in detail.[2] In brief, excitation was achieved using the output of a passively Q-switched, frequency tripled Nd:YAG laser (Teem Photonics, Powerchip NanoUV) producing pulses at 355 nm with a 500 Hz repetition rate, approximately 20 μJ energy, and 300 ps duration.

Data treatment: The pixel-to-wavelength conversion was done using a standard filter of Holmium oxide, which shows narrow bands in the UV-Vis spectral region. The global analysis was performed using a homemade written script in Matlab®.

Geometry optimizations of the ground and S_1 state, TD-DFT calculations, and SOC calculations.

Geometry optimizations were carried out in vacuum using the Gaussian 16, rev. A.03 software [3] with the hybrid B3LYP functional and the 6-311G(d,p)++ basis set. Time-dependent density functional theory (TD-DFT) calculations were performed at the same level of theory, using the lowest 30 singlets and 30 triplets. With these results, the energy diagrams and the theoretical electronic absorption spectra were constructed using a gaussian line broadening function with a half width at half maximum of 0.333 eV; obtaining a good agreement with the experimental absorption spectra, as shown in Figures S13 and S14.

For the relaxed potential energy surface scan in the ground state, we used Gaussian 16 with the keywords OPT=modredundant, D and S. The dihedral angle of the optimized ground state structure used as an input is shown in detail in Figure S12.

For calculating the charge density difference isosurfaces (CDD), we used the output from the TD-DFT calculations for singlet and triplet states and ran the calculation in Multiwfn software,[4] with a higher grid. The resulting *.cube files were then plotted in GaussView 6 to obtain the figures shown.

The S_1 excited state optimizations were carried out at the same level of theory using the optimized S_0 geometry as an initial guess. The resulting optimized geometries for S_o and S_1 were used to calculate the spin-orbit coupling (SOC) matrix elements, for which we used the ORCA 5.0 software[5] with the method of Zero-Order-Regular Approximation (ZORA)[6] to account for relativistic effects, at the B3LYP/6-311++G(d,p) level in a tight TD-DFT calculation with 30 states. We used a homemade script in Python to parse the SOC results.

II. Synthesis

2-bromo-3-methylpyridine 1-oxide (**5**)[7] and Et_3NBH_2CN[8] were prepared according to reported procedure.

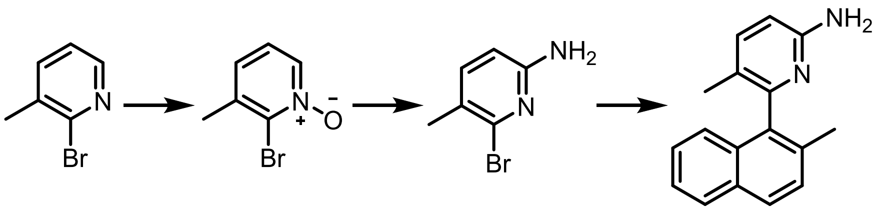

4 **5** **6** *rac*-**7**

2-Amino-6-bromo-5-methylpyridine (6)

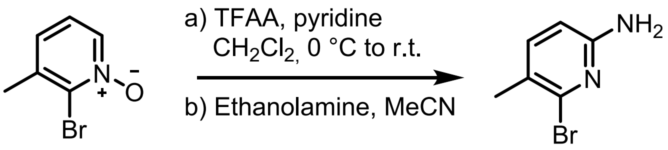

2-bromo-3-methylpyridine 1-oxide **5** (1.1 g, 5.85 mmol) was dissolved in 20 mL of dry CH_2Cl_2 and cooled to -5 °C in an ice/NaCl bath. Pyridine (0.95 mL, 11.7 mmol) was added followed by the dropwise addition of trifluoroacetic anhydride (1.22 mL, 8.77 mmol). The reaction was stirred for 15 min at low temperature then 6 hours at room temperature. Then, the solvent was evaporated followed by the addition of 12 mL of MeCN and 10 equiv. of ethanolamine (3.54 mL, 58.5 mmol). The reaction was stirred overnight at room temperature. After evaporating the crude mixture to dryness, the crude mixture was purified by flash column chromatography on silica gel (EtOAc:Pentane – 4:6) to obtain 810mg of **6** (74% yield) as a white precipitate (melting point: 91 – 93 °C).

^{1}H NMR (400 MHz, Methylene Chloride-d_2) δ 7.26 (d, J = 8.0 Hz, 1H), 6.39 (d, J = 8.1 Hz, 1H), 4.41 (s, 2H), 2.21 (s, 3H).

^{13}C NMR (101 MHz, $CDCl_3$) δ 156.6 (*C*), 141.9 (*C*), 140.7 (*C*H), 123.4 (*C*), 107.4 (*C*H), 20.8 (*C*H_3).

HRMS (ESI): Calculated for $C_6H_7BrN_2$ $[M+H]^+$: 186.9866 *m/z*; Found: 186.9860 *m/z*.

IR (neat, cm^{-1}) $\tilde{\nu}$ 3358, 3306, 30200, 2914, 2857, 1634, 1599, 1547, 1472, 1370, 1313, 1286, 1054.

2-Amino-5-methyl-6-(2-methylnaphthalen-1-yl)pyridin *rac*-7

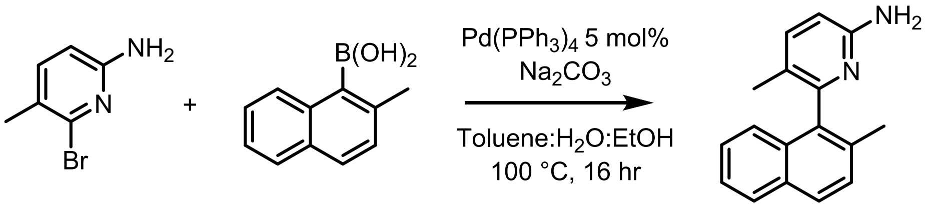

rac-**7**

In a Shlenck tube, 2-Amino-6-bromo-5-methylpyridine (250 mg, 1.34 mmol), (2-methylnaphthalen-1-yl)boronic acid (374 mg, 2.01 mmol), sodium carbonate (284 mg, 2.68 mmol), and $Pd(PPh_3)_4$ (78 mg, 0.067 mmol) were dissolved in degassed Toluene:H_2O:EtOH (7.5:1.5,:1.5 mL) solution mixture. The reaction mixture was stirred at 110 °C for 20 hours, and upon completion, it was extracted with EtOAc (3x10 mL). The organic layer was dried with Na_2SO_4, filtered, evaporated to dryness then purified by flash column chromatography on silica gel (EtOAc:Pentane – 4:6) to obtain 324 mg of **7** (97% yield) as a pale yellow precipitate (melting point: 167 – 170 °C).

^{1}H NMR (500 MHz, Methylene Chloride-d_2) δ 7.84 (d, *J* = 8.1 Hz, 1H), 7.79 (d, *J* = 8.4 Hz, 1H), 7.46 – 7.37 (m, 3H), 7.36 – 7.30 (m, 1H), 7.22 (d, *J* = 8.5 Hz, 1H), 6.54 (d, *J* = 8.3 Hz, 1H), 4.40 (br, 2H), 2.19 (s, 3H), 1.79 (s, 3H).

^{13}C NMR (126 MHz, Methylene Chloride-d_2) δ 157.3 (*C*), 155.9 (*C*), 140.3 (*C*H), 137.1 (*C*), 133.5 (*C*), 132.6 (*C*), 132.4 (*C*), 129.2 (*C*H), 128.4 (*C*H), 127.9 (*C*H), 126.5 (*C*H), 125.7 (*C*H), 125.3 (*C*H), 122.6 (*C*), 107.9 (*C*H), 19.9 (*C*H_3), 17.5 (*C*H_3).

HRMS (ESI): Calculated for $C_{17}H_{16}N_2$ $[M+H]^+$: 249.1387 *m/z*; Found: 249.1376 *m/z*.

IR (neat, cm^{-1}) $\tilde{\nu}$ 3281, 3149, 2916, 2852, 2380, 1618, 1565, 1507, 1479, 1413, 1374, 1343, 1266, 1106.

$[\alpha]_{20}^{D}$ for (+)- and (−)-**7** after CSP-HPLC resolution are +182 and −187 in CH_2Cl_2 (0.1 g/L)

Synthesis of 3

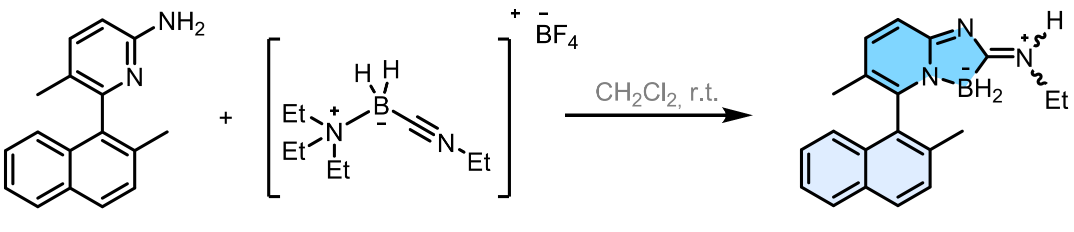

rac-**7**
(+)-(*P*)-**7**
(–)-(*M*)-**7**

rac-**3**
(+)-(*P*)-**3**
(–)-(*M*)-**3**

Et_3NBH_2CN (105 mg, 0.75 mmol) was dissolved in 5 mL dry CH_2Cl_2 followed by the addition of Et_3OBF_4 (143 mg, 0.75 mmol). The mixture was stirred overnight at room temperature under N_2 atmosphere. After evaporating the solvent, the mixture was washed with dry ether (3x5 mL) then dried under vacuum to obtain $[Et_3NBH_2CNEt]BF_4$ (**2**). Later on, **7** (124 mg, 0.5 mmol) was dissolved in 5 mL dry CH_2Cl_2 and added to $[Et_3NBH_2CNEt]BF_4$ under N_2 then stirred for 24 hours at room temperature. After the completion of the reaction as monitored by TLC and crude NMR, the solvent was evaporated and the crude mixture was purified by flash chromatography on silica gel (CH_2Cl_2:MeOH – 95:5) to obtain the racemic or enantiopure boramidine **3** (68-70% yield) as a white precipitate (melting point: 195 – 197 °C).

^{1}H NMR (400 MHz, Methylene Chloride-d_2) δ 7.95 – 7.88 (m, 2H, *major* + 0.25H *minor*), 7.85 (m, 1H, *major* + 0.25H, *minor*), 7.71 (d, *J* = 8.5 Hz, 0.25H, *minor*) 7.50 – 7.43 (m, 2H, *major*, + 0.5H, *minor*), 7.42 – 7.32 (m, 1H, *major* + 0.25H, *minor*), 7.29 (d, *J* = 8.6 Hz, 1H, *major*), 7.22 (d, *J* = 8.6 Hz, 0.25H, *minor*), 7.10 (d, *J* = 8.4 Hz, 0.25H, *minor*) 7.05 (d, *J* = 8.6 Hz, 1H, *major*), 3.49 (m, 0.5H. *minor*), 3.18 – 3.09 (m, 2H, *major*), 2.17 (s, 3H, *major*), 1.87 (s, 3H, *major*), 1.80 (s, 0.7H, *minor*), 1.19 (t, *J* = 7.3 Hz, 0.7H, *minor*), 1.11 (t, *J* = 7.3 Hz, 3H, *major*).

^{13}C NMR (126 MHz, CD_2Cl_2) δ 159.7 (*C*), 151.3 (*C*), 144.2 (*C*H, *major*), 143.2 (*C*H, *minor*), 134.4 (*C*), 134.2 (*C*), 132.6 (*C*), 131.0 (*C*), 130.7 (*C*), 129.9 (*C*H, *major*), 129.3 (*C*H, *minor*), 129.2 (*C*H, *major*), 128.9 (*C*H, *major*), 128.7 (*C*H, *minor*), 127.6 (*C*H, *major*), 127.2 (*C*H, *minor*), 126.4 (*C*), 126.1 (*C*H, *major*), 125.8 (*C*H, *minor*), 124.6 (*C*H, *minor*), 124.2 (*C*H, *major*), 114.3 (*C*H, *minor*), 112.3 (*C*H, *major*), 42.2 (CH_2, *major*), 37.5 (CH_2, *minor*), 19.7 (CH_3, *major*), 19.6 (CH_3, *minor*), 17.4 (CH_3, *minor*), 17.3 (CH_3, *major*), 15.0 (CH_3, *minor*), 14.9 (CH_3, *major*).

^{11}B NMR (128 MHz, CD_2Cl_2) δ -13.6, m.

HRMS (ESI): Calculated for $C_{20}H_{22}BN_3$ $[M+H]^+$: 316.1984 *m/z*; Found: 316.1988 *m/z*.

IR (neat, cm^{-1}) $\tilde{\nu}$ 3222, 3183, 3051, 2973, 2924, 2857, 2380, 1623, 1582, 1471, 1453, 1375, 1314, 1258, 1226, 1125, 1051.

$[\alpha]_{20}^{D}$ for (+)- and (−)-**3** are +237 and −240 in CH_2Cl_2 (0.1 g/L)

III. (Chir)Optical Measurements

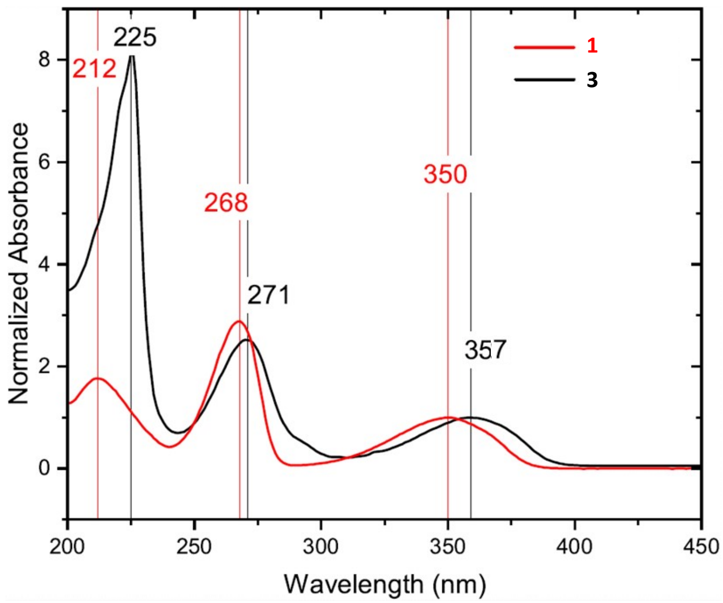

Figure S1. Normalized absorption spectra of **1** (red) and **3** (black) in ACN.

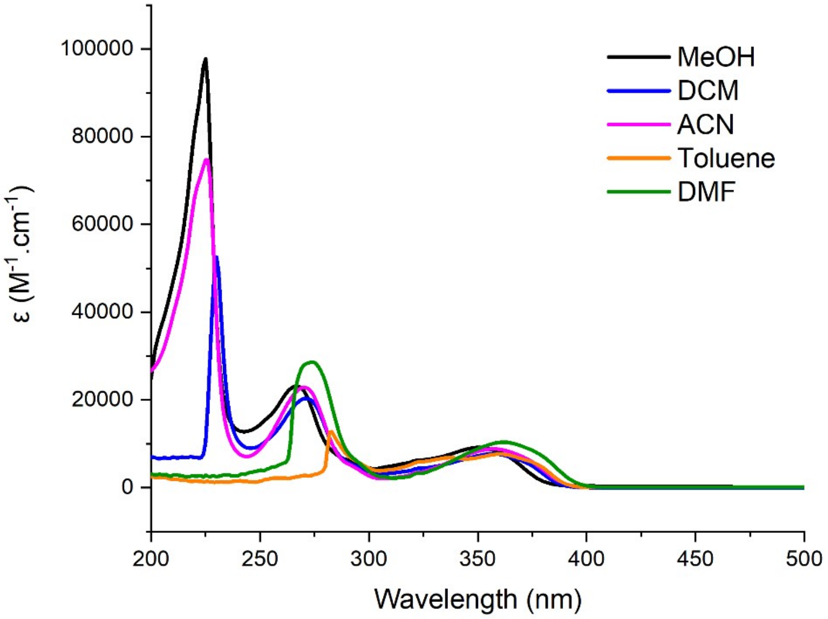

Figure S2. Electronic absorption spectrum of **3** in different solvents (2.10^{-5} M).

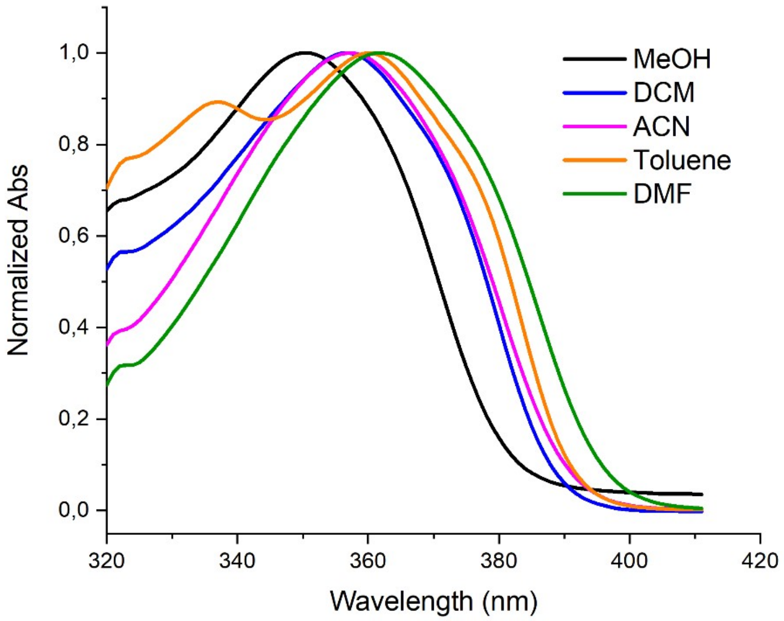

Figure S3. Normalized electronic absorption spectrum of **3** (region 320-420 nm) in different solvents (2.10^{-5} M).

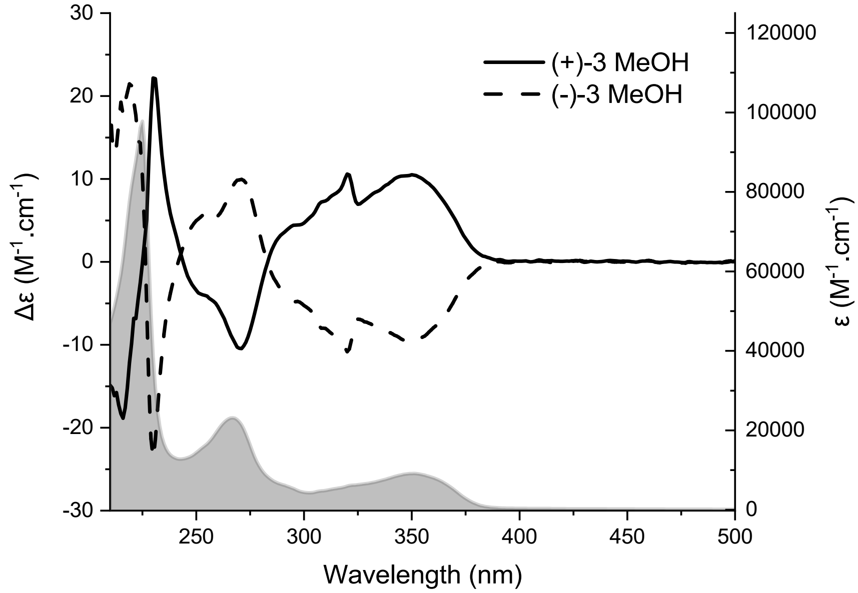

Figure S4. Electronic absorption and ECD spectra of **3** in MeOH (2.10^{-5} M).

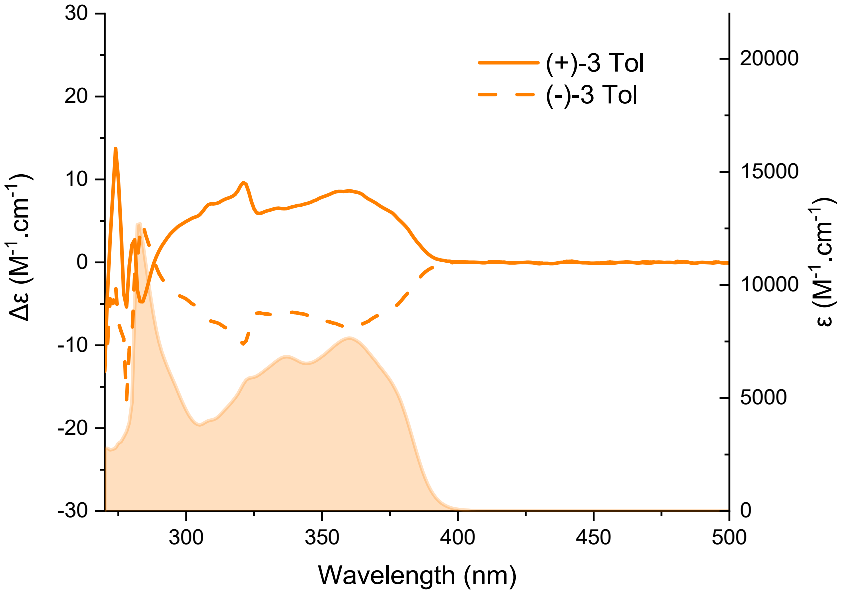

Figure S5. Electronic absorption and ECD spectra of **3** in toluene (2.10^{-5} M).

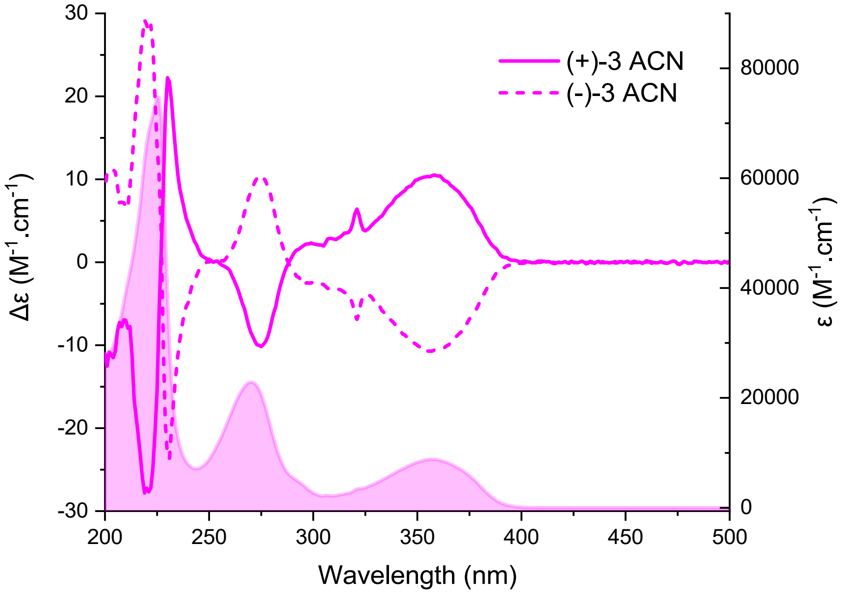

Figure S6. Electronic absorption and ECD spectra of **3** in ACN (2.10^{-5} M).

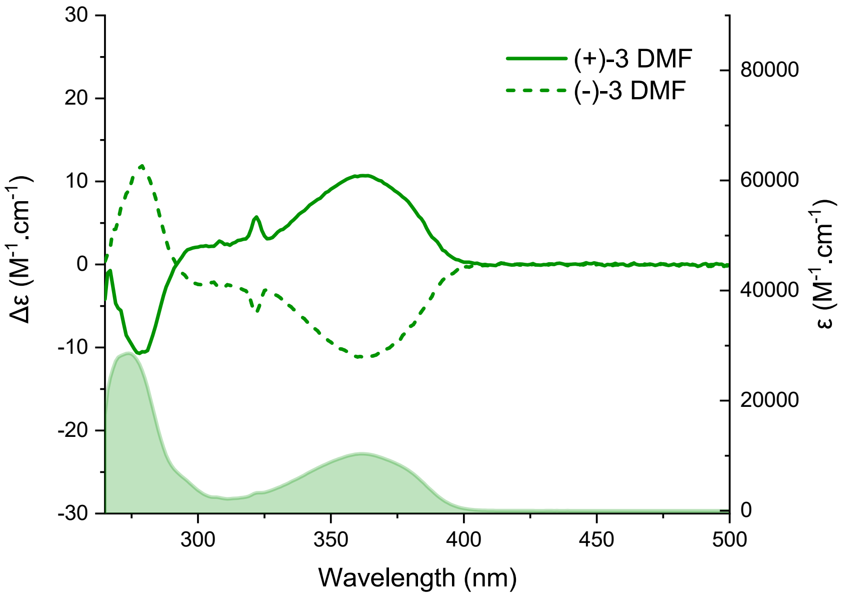

Figure S7. Electronic absorption and ECD spectra of **3** in DMF (2.10^{-5} M).

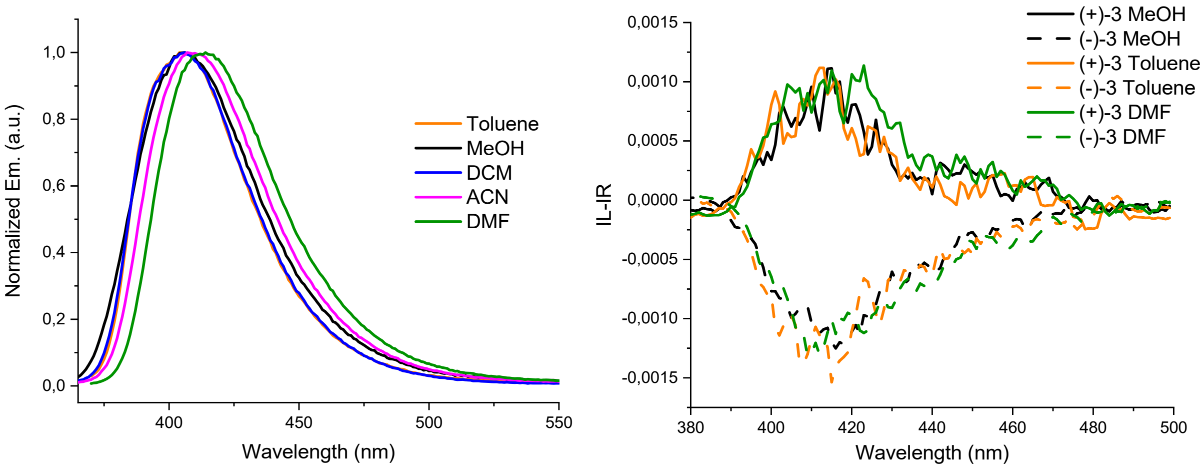

Figure S8. Normalized emission spectra (Left) and CPL spectra (right) of **3** in different solvents.

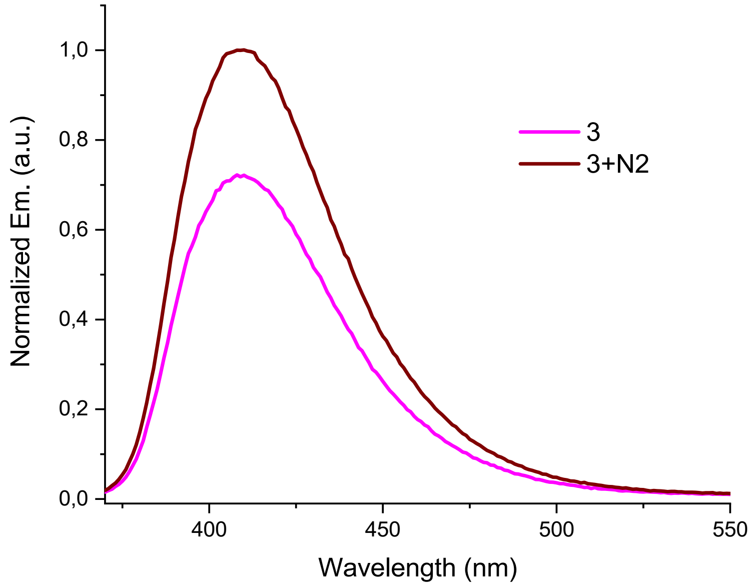

Figure S9. Normalized emission spectra of **3** in ACN solution not saturated (magenta) and saturated (brick) with N_2.

Table S1. Photophysical data of 3[a]

Solvent	λ_{max} (nm)	ε (M^{-1} cm^{-1})[b]	λ_{em} (nm)	Stokes shift (cm^{-1})	FWHM cm^{-1}	FWHM (nm)
MeOH	350	9135	406	3940	3210	54
DCM	357	7720	406	3381	2986	50
ACN	357	8820	407	3441	3031	52
Tol	360	7650	404	3025	2986	50
DMF	362	10360	414	3470	3024	53

[a] Concentrations $2 \cdot 10^{-5}$ M; [b] calculated for the most red-shifted band 350-362 nm;

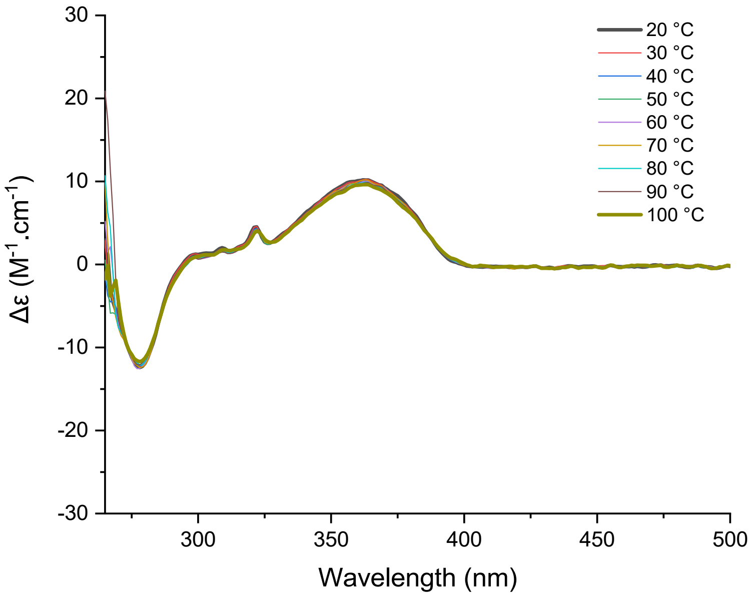

Figure S10. Variable Temperature ECD spectra of **3** from 20 °C to 100 °C in DMF

IV. Time-Resolved Fluorescence

Table S2. Fluorescence lifetimes determined by Time-Correlated Single-Photon Counting (TCSPC). Error on the lifetime: ±2%.

**Compound, solvent, condition**	**t (ns)**
**1**, ACN, N_2	9.4
**1**, ACN, Air	8.4
**1**, TOL, N_2	7.0
**1**, TOL, Air	6.6
**3**, ACN, N_2	6.7
**3**, ACN, Air	5.9
**3**, TOL, N_2	5.3
**3**, TOL, Air	5.0

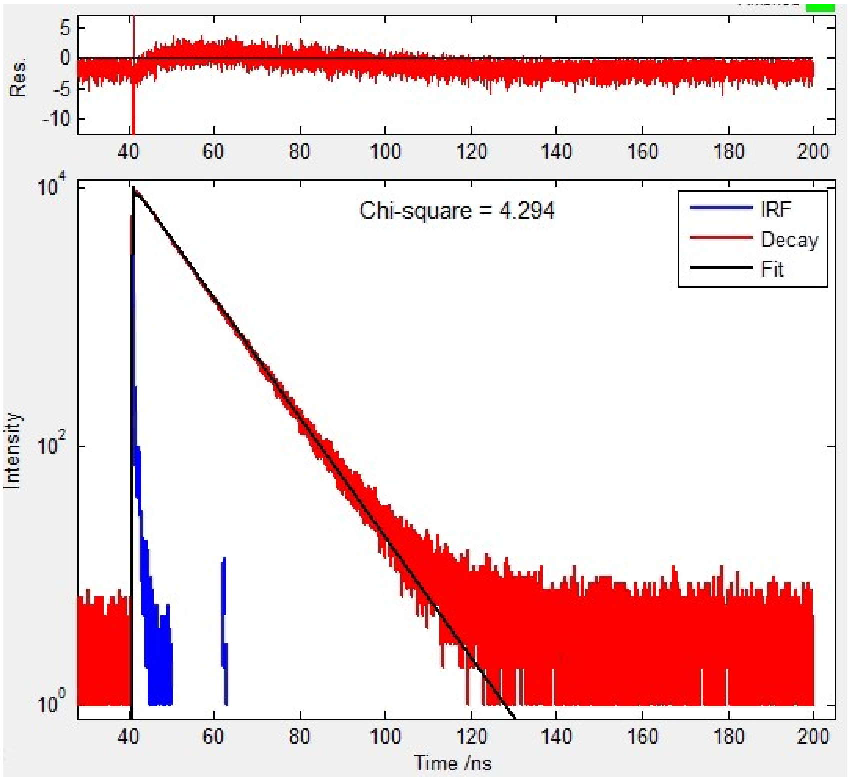

Figure S11. Experimental fluorescence decay measured with **1** in ACN under nitrogen flow (red), instrument response function (blue) and best single-exponential fit (black).

V. Transient Absorption Spectra

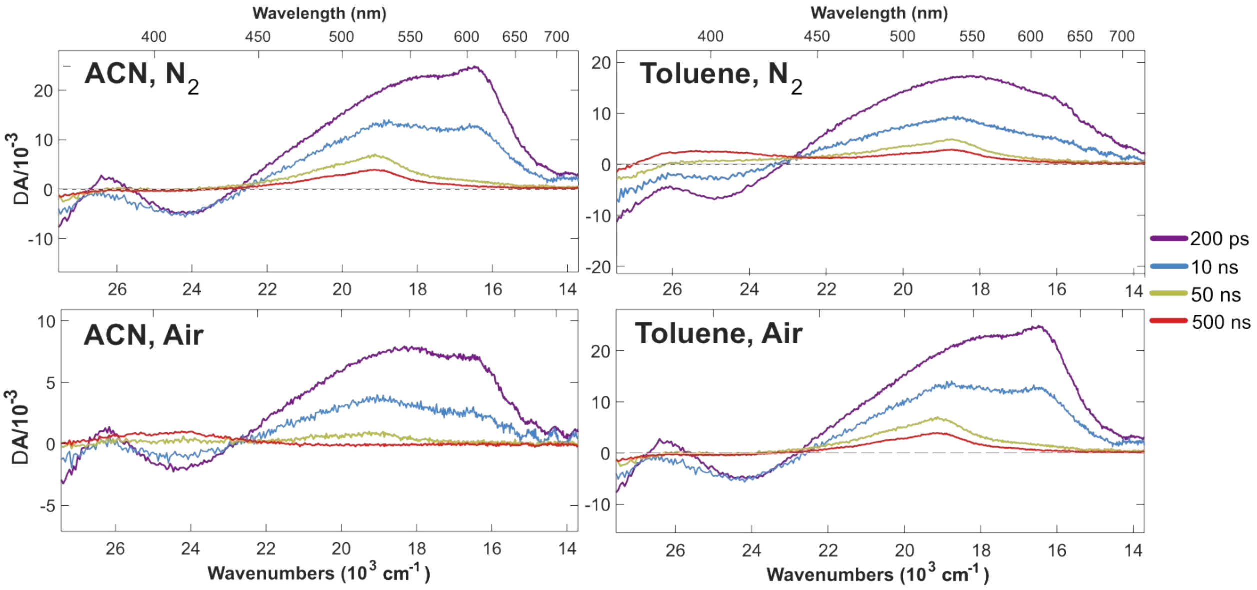

Figure S12. Transient absorption spectra measured after 355 nm excitation of **1** in ACN (left) and toluene (right), under nitrogen (upper) and air flow (bottom).

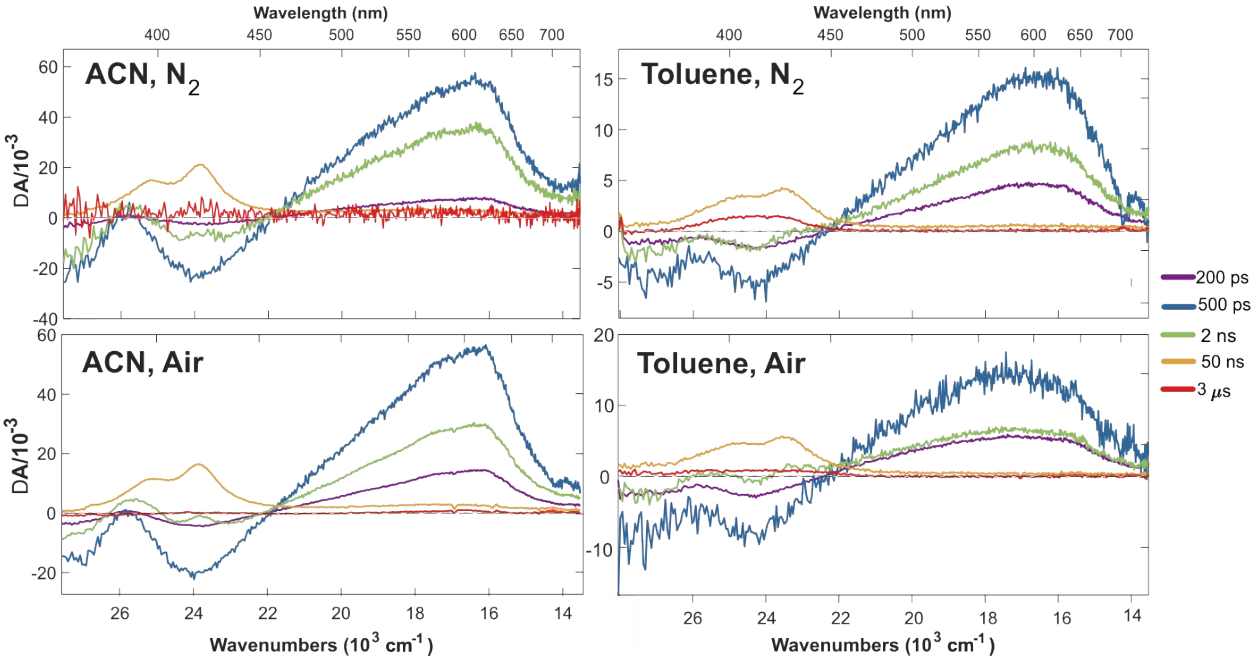

Figure S13. Transient absorption spectra measured after 355 nm excitation of **3** in ACN (left) and toluene (right), under nitrogen (upper) and air flow (bottom).

VI. Quantum-Chemical Calculations

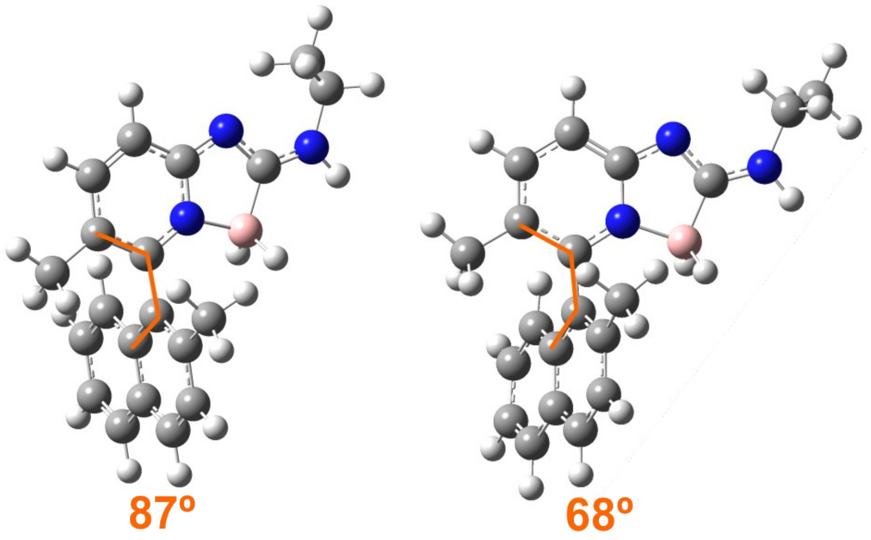

Figure S14. Optimized structures of **3** in the ground (left) and excited (right) states. The dihedral angle (D) is highlighted in orange.

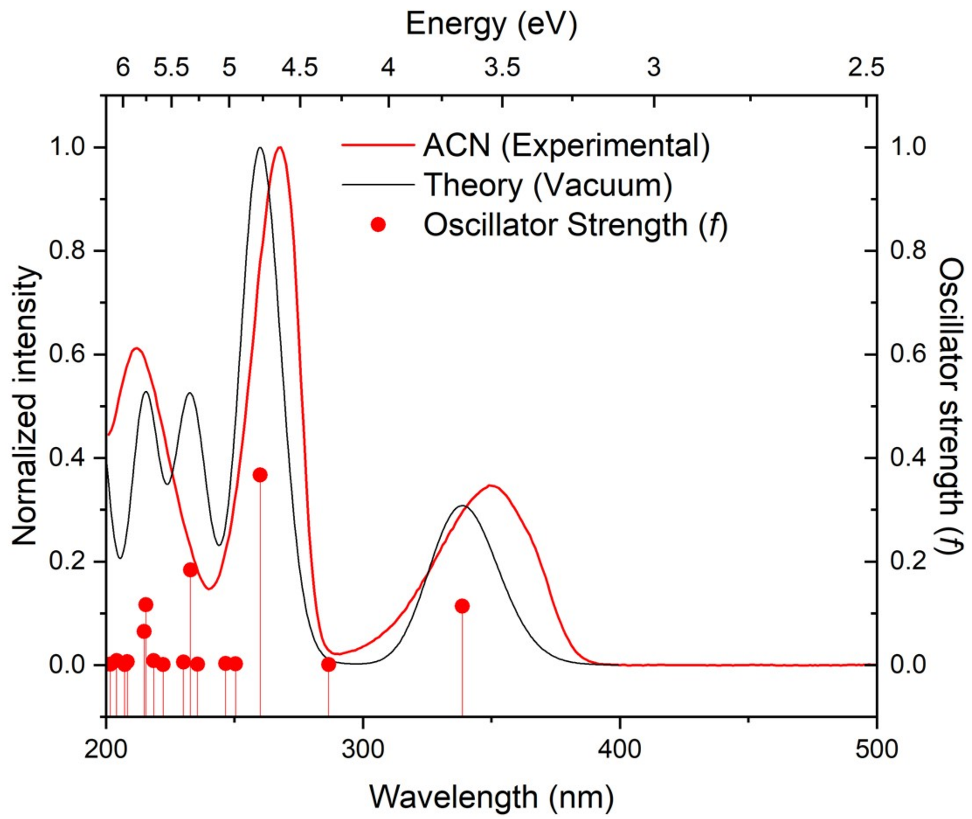

Figure S15. Comparison between the experimental (ACN) and calculated (vacuum) electronic absorption spectra of **1**. The calculated oscillator strength is also shown.

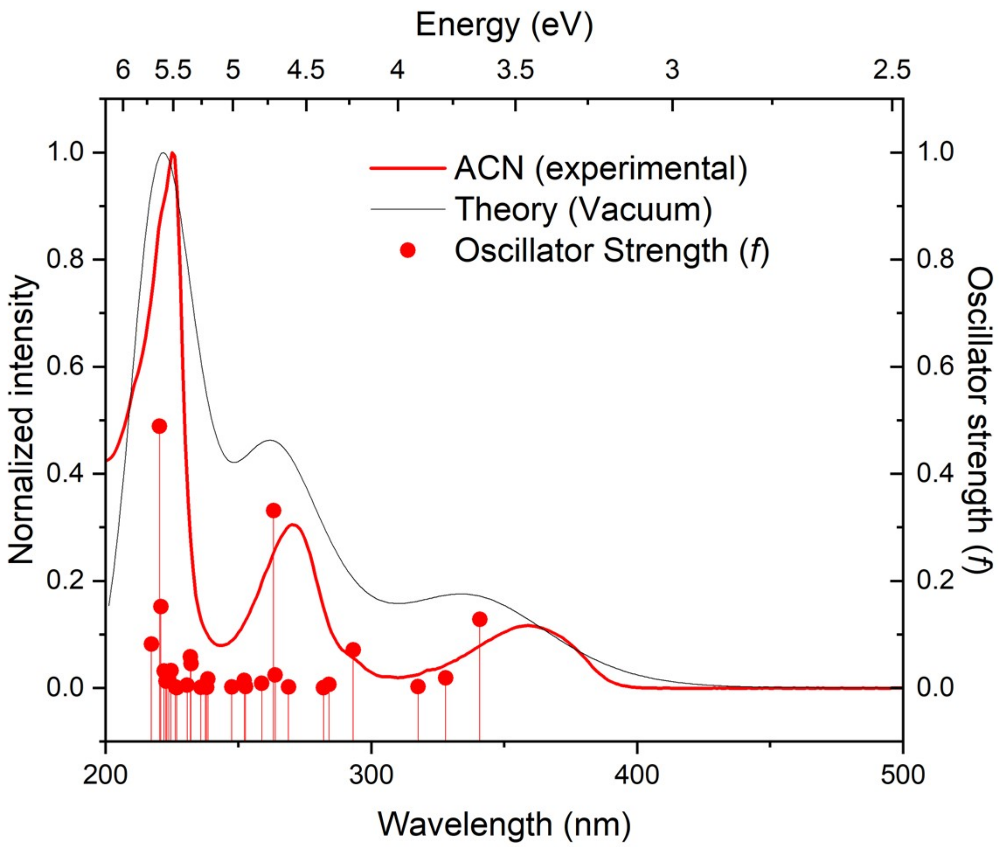

Figure S16. Comparison between the experimental (ACN) and calculated (vacuum) electronic absorption spectra of **3**. The calculated oscillator strength is also shown.

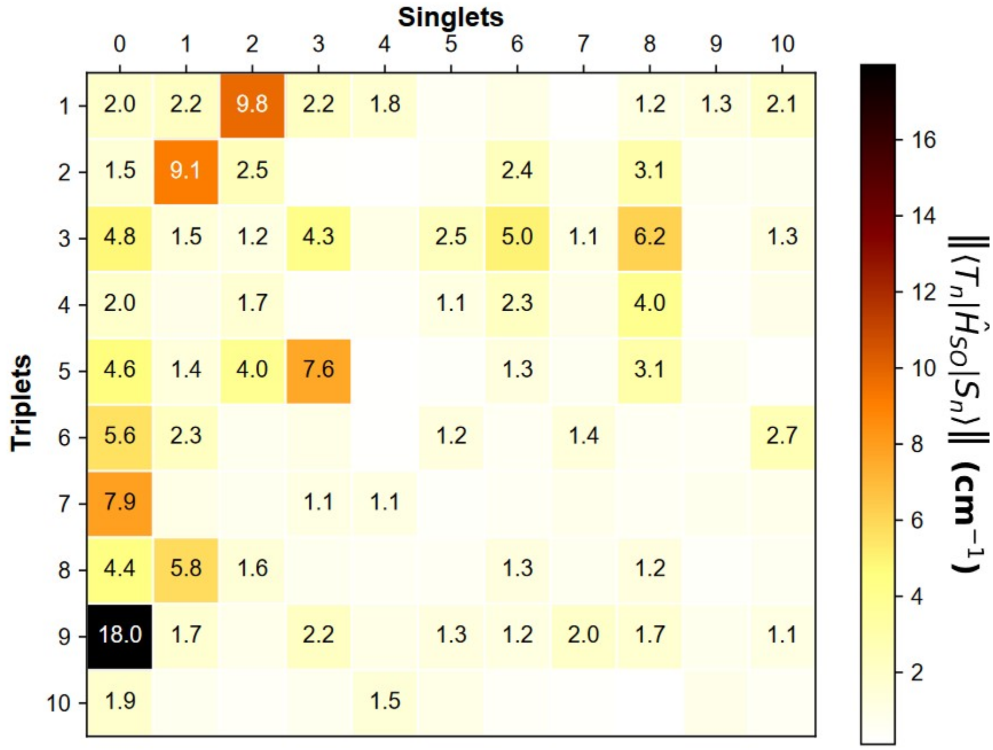

Figure S17. *Magnitude of the spin-orbit coupling (SOC) matrix element between the first 10 singlets and triplet states of* ***1****, calculated from the optimized S_1 state.*

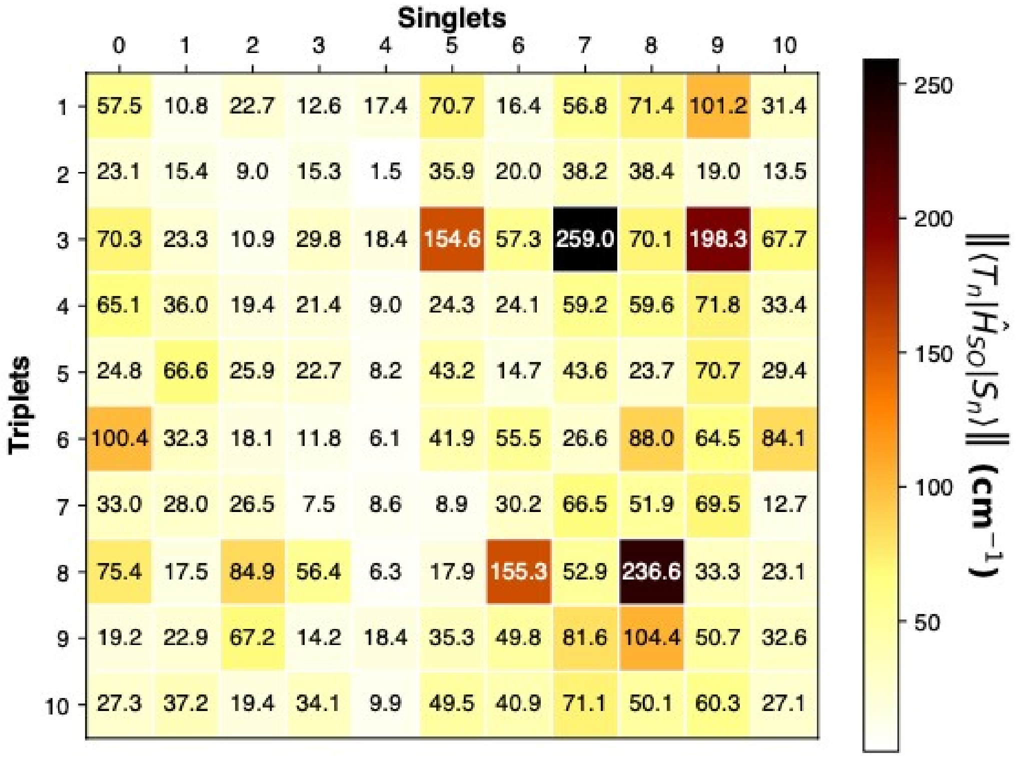

Figure S18. Magnitude of the spin-orbit coupling (SOC) matrix element between the first 10 singlets and triplet states of **3**, calculated from the optimized S_1 state.

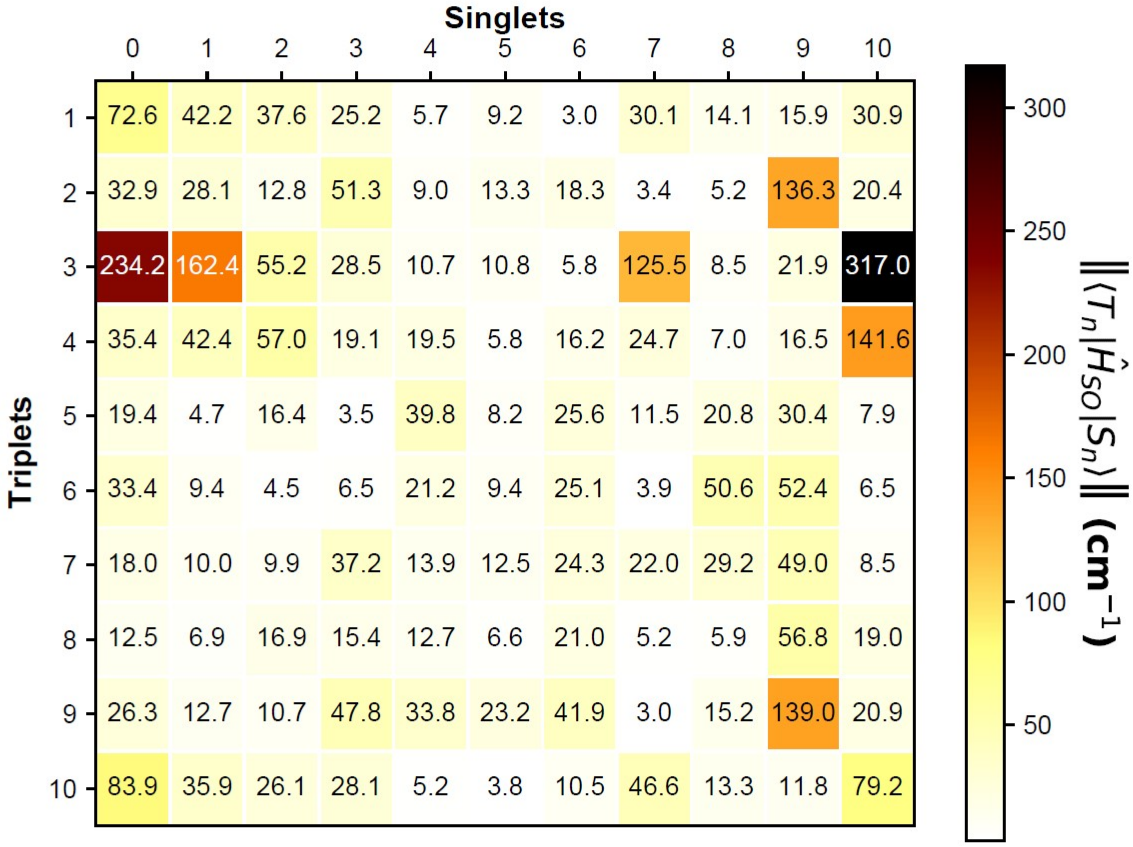

Figure S19. Magnitude of the spin-orbit coupling (SOC) matrix element between the first 10 singlets and triplet states of **3**, calculated from the optimized S_0 state.

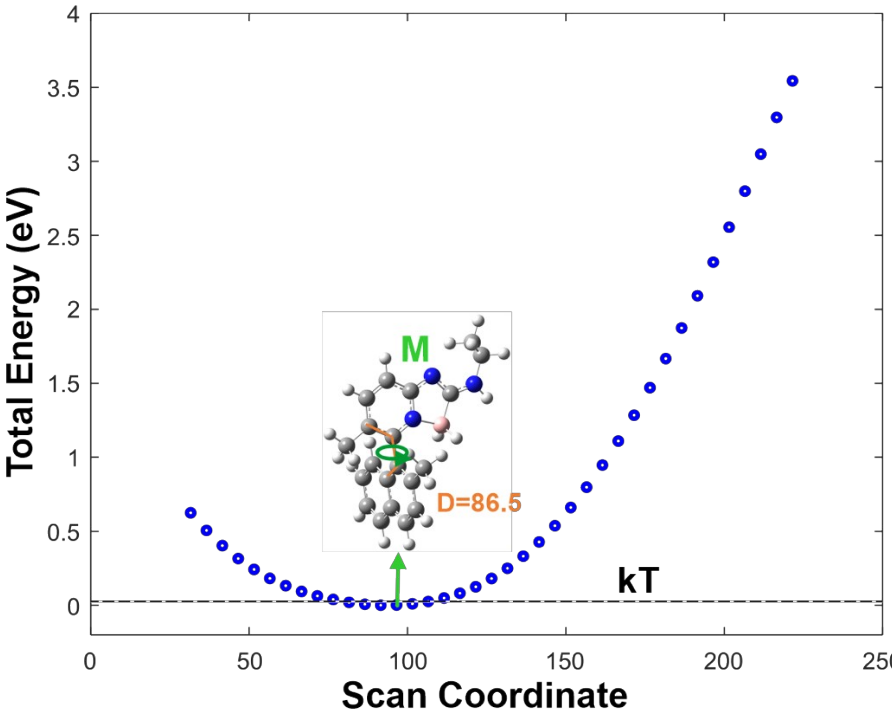

Figure S20. Ground-state energy of **3** as a function of the dihedral angle D. The dashed line indicates the value of kT at room temperature

Table S3. Magnitude of the Spin-orbit coupling (SOC) constant of **3** as a function of the dihedral angle D. It calculated from the optimized structure in the S_1 with the dihedral angle D at fixed values. The values at the minimum S_0 structure are in green.

Dihedral angle (degrees)	S_1-T_1 (cm^{-1})	S_1-T_2 (cm^{-1})	S_1-T_3 (cm^{-1})
10	2.9	5.2	3.5
20	4.0	10.6	10.3
30	5.4	19.7	19.7
45	2.7	36.8	35.4
60	10.4	50.9	7.4
70	29.8	68.1	18.5
80	52.6	69.6	105.1
**87**	42.2	28.1	162.4
90	45.4	13.1	178.3
111	89.9	17.3	237.7

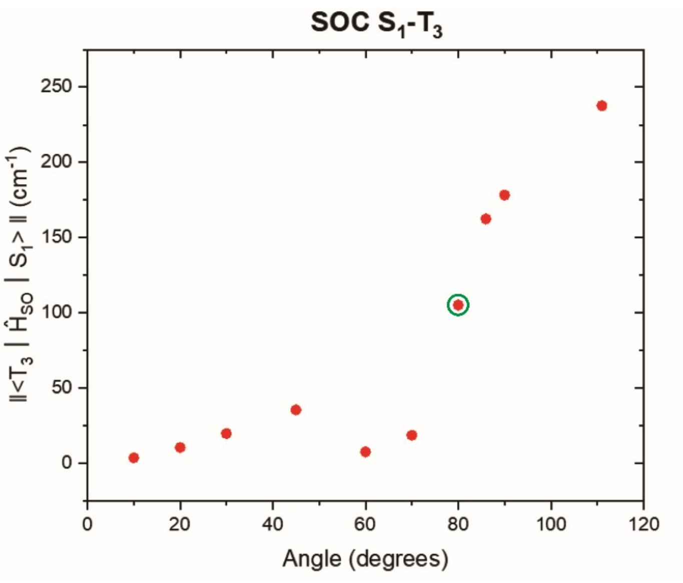

Figure S21. Graphic representation of the SOC matrix element vs dihedral angle for the S_1-T_3 states of **3**. The most stable S_0 structure is highlighted in green.

Cartesian Coordinates of **1**

0 1

C	-2.58060400	1.06167200	0.22355100
C	-3.52272600	0.05509800	0.19248200
C	-3.08346900	-1.26634200	-0.00875900
C	-0.81521500	-0.47959700	-0.13112500
C	-1.74110300	-1.54115400	-0.17006800
H	-3.80594300	-2.07449500	-0.03714600
H	-1.37050900	-2.54513500	-0.32661100
N	-1.27235400	0.79867200	0.06650900
C	1.06024500	0.62195900	-0.18014200
H	2.73407100	1.72368900	-0.19165300
N	2.38280100	0.78523700	-0.30182500
C	3.35147400	-0.29393000	-0.49504000
H	4.16434300	0.09738800	-1.11345600
H	2.84979200	-1.08080700	-1.05859900
C	3.89942100	-0.85313900	0.81995600
H	3.08996200	-1.26776600	1.42387600
H	4.62237000	-1.64949500	0.61990700
H	4.40350700	-0.07690200	1.40282100
H	-0.11050300	2.57134000	-0.84123600
H	0.08239800	2.32381200	1.13951000
B	-0.02908600	1.77767200	0.06660900
N	0.53096000	-0.60222500	-0.27183900
H	-2.84117800	2.10203300	0.37384800
H	-4.57086100	0.28877200	0.32065300

Cartesian Coordinates of **3**

0 1

C	2.93129400	0.15071200	-2.35057500
C	3.65931100	-0.80266900	-1.68658200
C	3.30805700	-1.19536100	-0.37157000
C	2.17729200	-0.57958300	0.25163100
C	1.44067700	0.41341800	-0.46744200
C	1.80757300	0.77896900	-1.75374300
H	4.90001300	-2.63815800	-0.14471400
H	3.21352400	0.43989300	-3.35747200
H	4.51648300	-1.26747800	-2.16282800
C	4.04435600	-2.17879200	0.33956300
C	1.83028300	-0.98887900	1.56804900
C	2.56421600	-1.94429900	2.23023600
C	3.68459400	-2.54662400	1.61306400
H	0.96735500	-0.54535200	2.05015000
H	2.27899000	-2.24392200	3.23255300
H	4.25348900	-3.29996900	2.14615500
C	0.27580200	1.08039000	0.19237500
C	0.43342800	2.20862400	0.99748900
C	-0.72775900	2.76847800	1.56430400
C	-2.08856900	1.09242000	0.52881900
C	-1.97818200	2.23001700	1.34294500
H	-0.63042000	3.64817400	2.19284500
H	-2.87548600	2.65174100	1.77507700
C	1.03268000	1.80727400	-2.54236200
H	0.07328200	1.39708000	-2.87324600
H	0.81404200	2.69868300	-1.94881000
H	1.58899500	2.11798400	-3.42864600
C	1.79286000	2.81412500	1.25284500
H	2.46812800	2.10419900	1.73840500
H	2.27776900	3.13086000	0.32494100
H	1.70437100	3.68921000	1.89970200
N	-0.95294200	0.55446000	-0.02370200
C	-2.95029400	-0.58109800	-0.56094200
H	-3.63741300	-2.16618500	-1.57932600
N	-3.92454700	-1.38002200	-1.01762600
C	-5.34230200	-1.25361200	-0.68305900
H	-5.91944500	-1.58101400	-1.55275100
H	-5.54375400	-0.19351300	-0.52682900
C	-5.74319700	-2.05473800	0.55805100
H	-5.18600900	-1.70815300	1.43058300
H	-6.81136100	-1.93091900	0.76003800
H	-5.54562400	-3.12207800	0.42303300
H	-1.11798000	-0.56298400	-2.04657600
H	-0.88582400	-1.72106100	-0.43322700
B	-1.38288600	-0.71469000	-0.87749700
N	-3.25128500	0.44896100	0.23130800

VII. CSP-HPLC Resolution

20 mg of *rac*-**7** were dissolved in 1.5 ml DCM. 500 µL of this solution were injected in Semipreparative CHIRALPAK® IC column using mixture of Hexane(+0.1% Et_2NH):iPrOH, 95:5 as an eluent and injection flow rate of 4 mL/min.

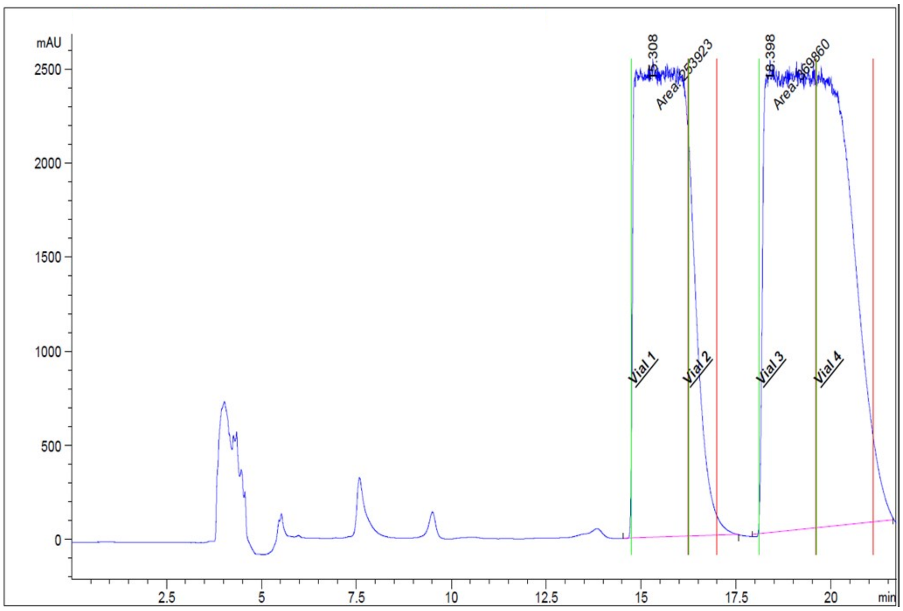

Figure S22. Semipreparatice CSP-HPLC resolution of rac-**3**.

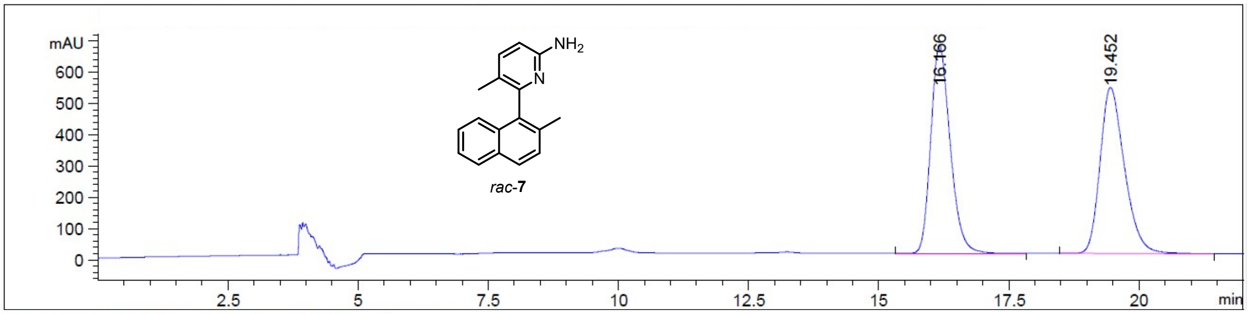

Signal 1: DAD1 A, Sig=254,4 Ref=off

Peak #	RetTime [min]	Type	Width [min]	Area [mAU*s]	Height [mAU]	Area %
1	16.166	BB	0.3990	1.72513e4	664.23041	49.9053
2	19.452	BBA	0.5012	1.73167e4	529.96576	50.0947

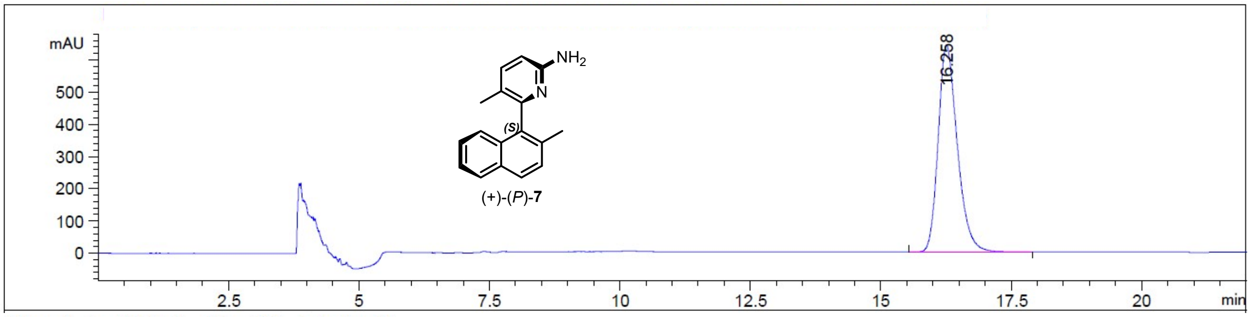

Signal 1: DAD1 A, Sig=254,4 Ref=off

Peak #	RetTime [min]	Type	Width [min]	Area [mAU*s]	Height [mAU]	Area %
1	16.258	BB	0.3855	1.63663e4	641.85016	100.0000

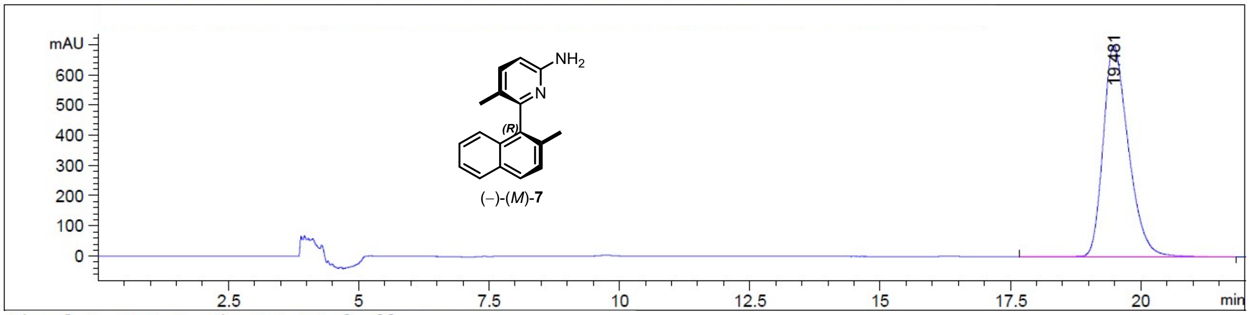

Signal 1: DAD1 A, Sig=254,4 Ref=off

Peak #	RetTime [min]	Type	Width [min]	Area [mAU*s]	Height [mAU]	Area %
1	19.481	BBA	0.5044	2.35506e4	700.12549	100.0000

Figure S23. Chromatograms of rac-**3** (Top), and (+)-, and (-)-**3** (middle and bottom) after resolution.

VIII. Crystallographic Data

All data were collected on an XtaLAB Synergy-S diffractometer equipped with a hypix arc 150 detector, using Cu Kα radiation. Data reduction was carried out in the crysalis Pro Software.[9] Structure solution was made using dual space methods in the shelxt program[10] Refinements were carried out in ShexlL[11] within the Olex2[12] software.
The resonant scattering is weak for this sample (Friedif 12) as reflected by the large error of the refined Flack parameter (-0.1(4)). However, all indicators confirm the absolute structure. The Hooft parameter is 0.05(6) with probability of the absolute structure being true P3(true) equal to 1 and the post refinement Parsons-Flack x determined using 2246 quotients is −0.03(7)
Details for the refinement for the structure can be found in the table below, with a representation of the asymmetric unit with displacement ellipsoids drawn at 50 percent probability.

Crystallization procedure for (−)-(*M*)-**7**: Slow evaporation of a deuterated dichloromethane solution of (−)-(*M*)-**7** resulted in the growth of mono crystals.

Table S4. Crystal data and structure refinement for (−)-(*M*)-**7**.

**CCDC**	**2321027**	
Empirical formula	C17 H16 N2	
Formula weight	248.32	
Temperature	100.00(11) K	
Wavelength	1.54184 Å	
Crystal system	Orthorhombic	
Space group	$P2_12_12$	
Unit cell dimensions	a = 25.8699(2) Å	a= 90°
	b = 14.53966(15) Å	b= 90°
	c = 7.44906(8) Å	g = 90°
Volume	2801.89(5) Å^3	
Z	8	
Density (calculated)	1.177 Mg/m^3	
Absorption coefficient	0.538 mm^{-1}	
F(000)	1056	
Crystal size	0.158 x 0.071 x 0.031 mm^3	
Theta range for data collection	3.417 to 73.875°.	
Index ranges	-31<=h<=32, -17<=k<=14, -9<=l<=9	
Reflections collected	85307	
Independent reflections	5555 [R(int) = 0.0335]	
Completeness to theta = 67.684°	100.0 %	
Absorption correction	Gaussian	
Max. and min. transmission	1.000 and 0.844	
Refinement method	Full-matrix least-squares on F^2	

Data / restraints / parameters	5555 / 0 / 364
Goodness-of-fit on F^2	1.074
Final R indices [I>2sigma(I)]	R1 = 0.0292, wR2 = 0.0752
R indices (all data)	R1 = 0.0301, wR2 = 0.0759
Absolute structure parameter	-0.03(7)
Extinction coefficient	0.00139(18)
Largest diff. peak and hole	0.144 and -0.121 e.Å^{-3}
Flack parameter	-0.03(7)

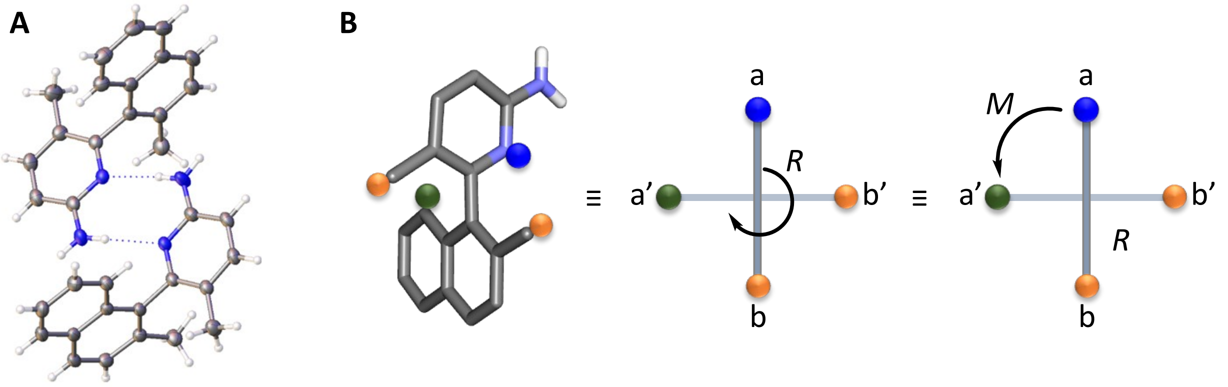

Figure S24. **A**). View of the asymmetric unit of (−)-(*M*)-**7**. Displacement ellipsoids are drawn at the 50 percent probability level; **B**) Schematic representation of the absolute configuration.

IX. NMR Spectra

Enantiodifferentiation of *rac*-3 and separated enantiomers

^{1}H NMR (400 MHz, toluene-d_8) studies were done in NMR tube, 1 mg of **3** and 1 equiv. of [Bu$_4$N][Δ-BINPHAT] in 0.6 mL of toluene-d_8.

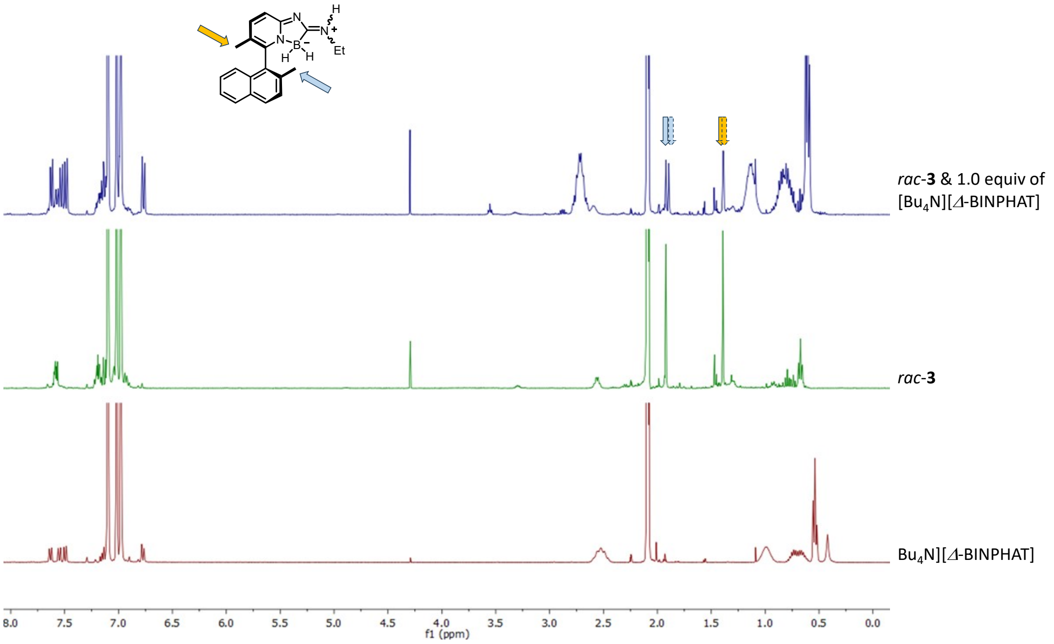

Figure S25. Full NMR spectra of [Bu$_4$N][Δ-BINPHAT] (brown), *rac*-**3** (green) and 1:1 mixture of [Bu$_4$N][Δ-BINPHAT] and rac-**3** in toluene-d_8.

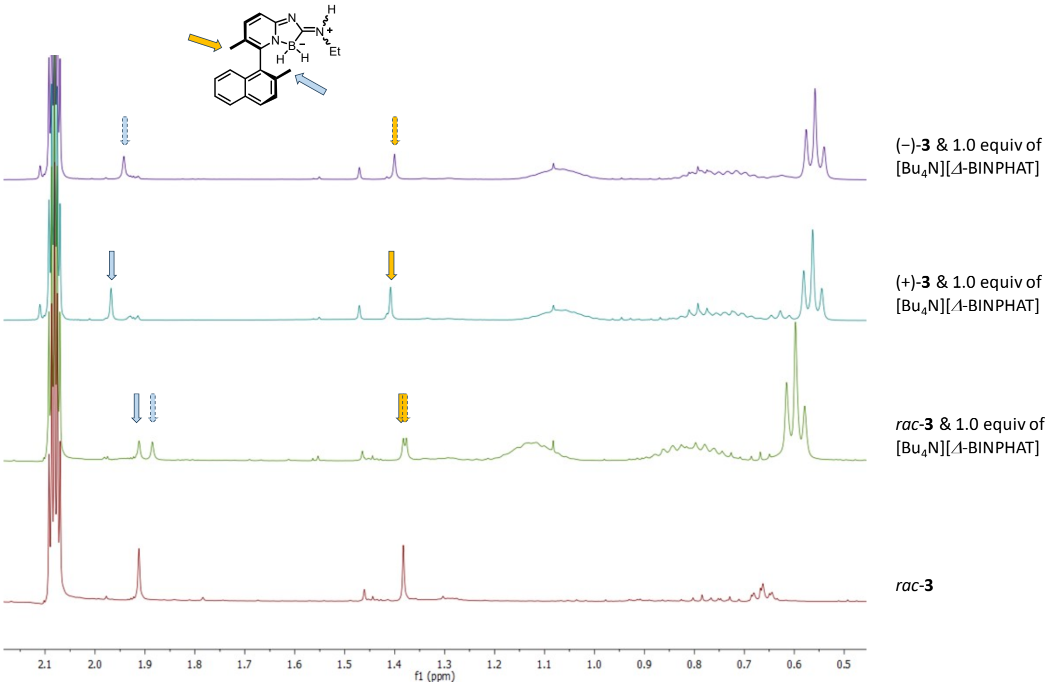

Figure S26. Zoomed region of NMR spectra of *rac*-**3** (brown), 1:1 mixture of [Bu$_4$N][Δ-BINPHAT] and *rac*-**3** (green), 1:1 mixture of [Bu$_4$N][Δ-BINPHAT] and (+)-**3** (cyan), and 1:1 mixture of [Bu$_4$N][Δ-BINPHAT] and (−)-**3** (navy) in toluene-d_8.

NMR Spectra of Synthesized Products

1. Compound **6**: ^{1}H NMR (400 MHz) in CD_2Cl_2; ^{13}C and ^{13}Cdept (101 MHz) NMR in $CDCl_3$

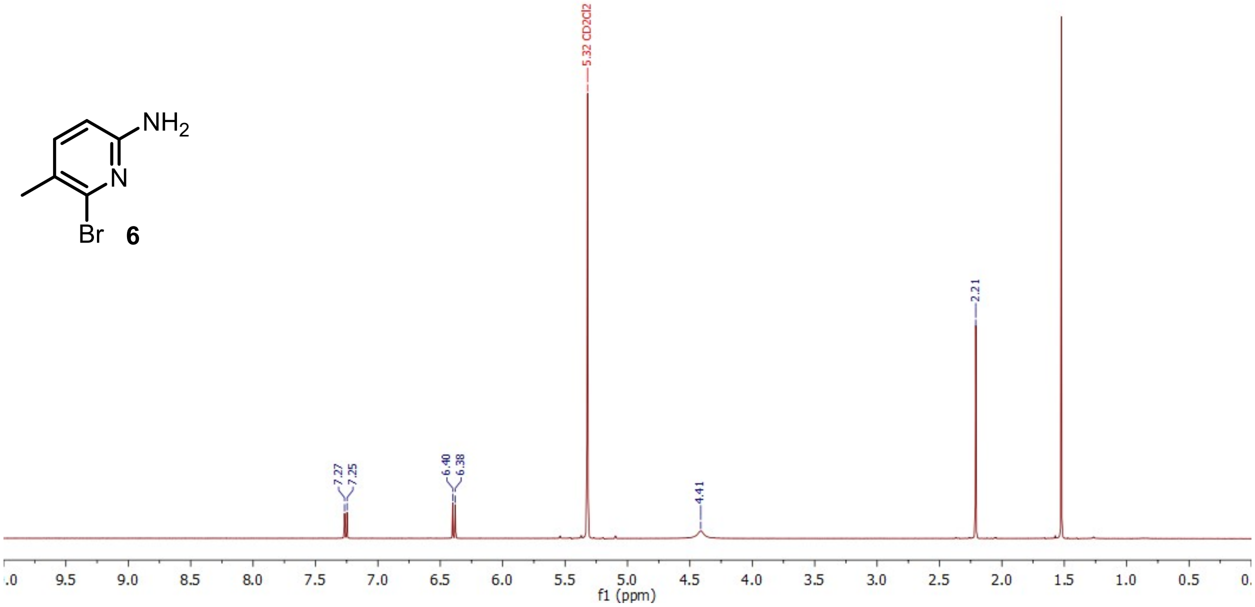

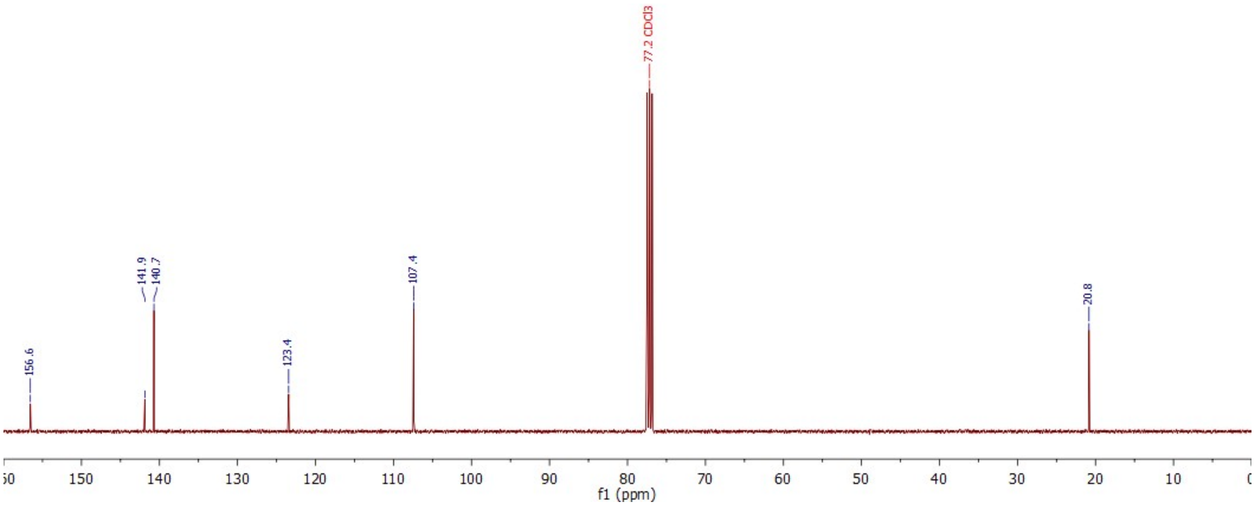

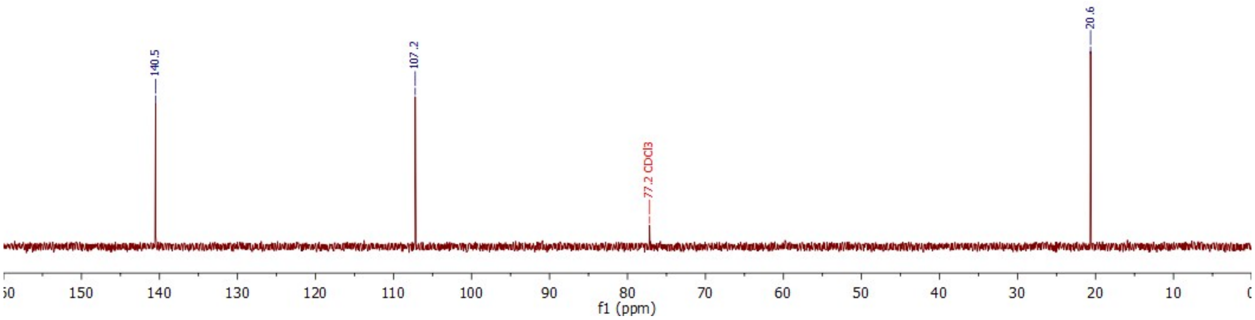

2. Compound **7:** ^{1}H NMR (500 MHz), ^{13}C and ^{13}Cdept NMR (126 MHz) in CD_2Cl_2

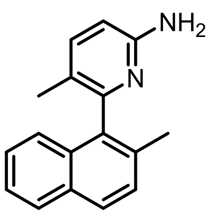

7

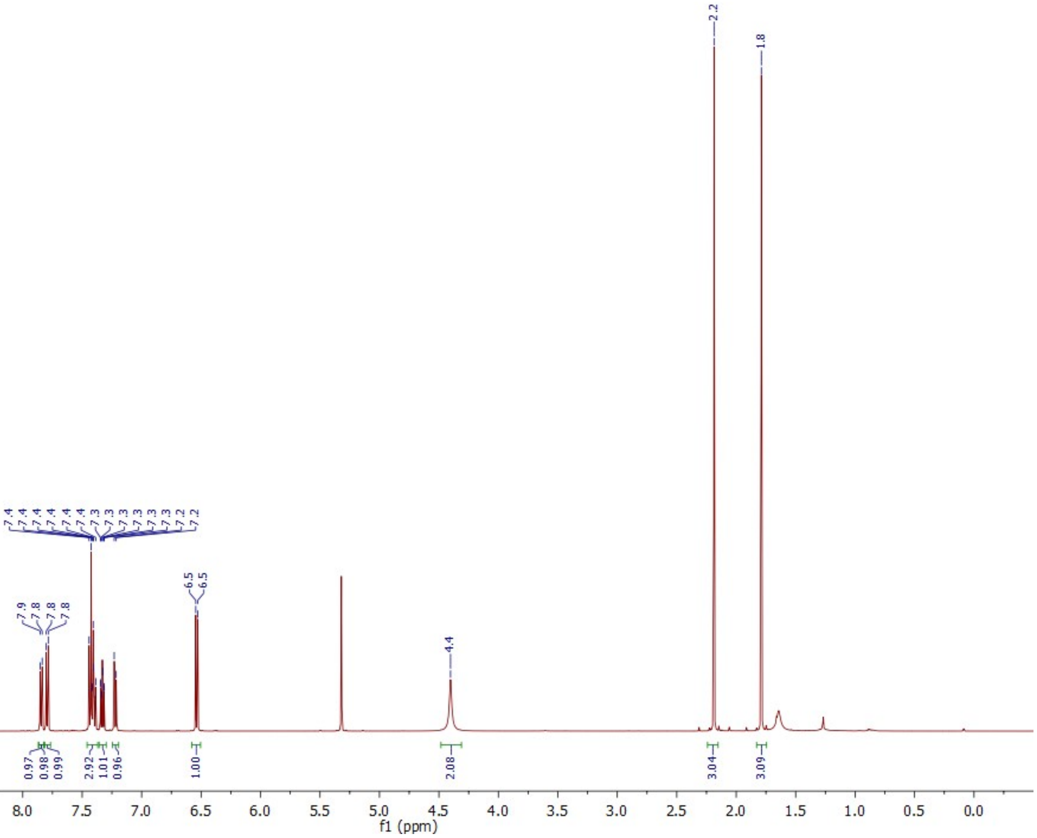

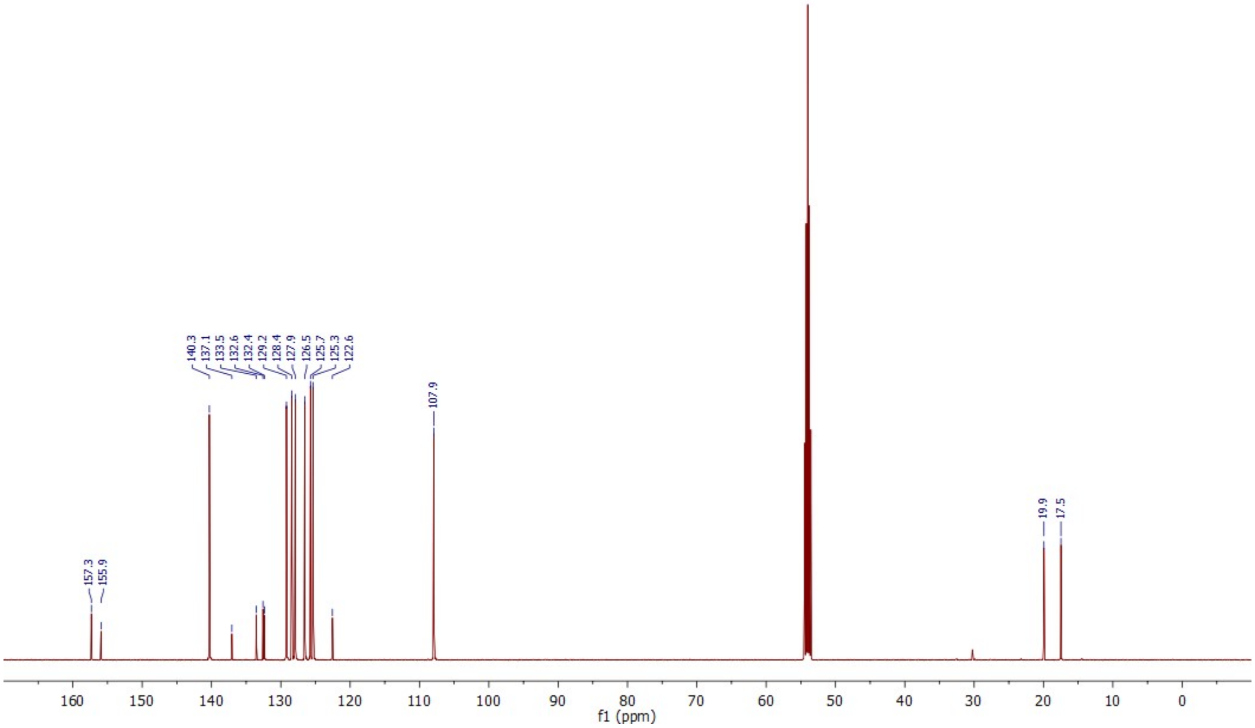

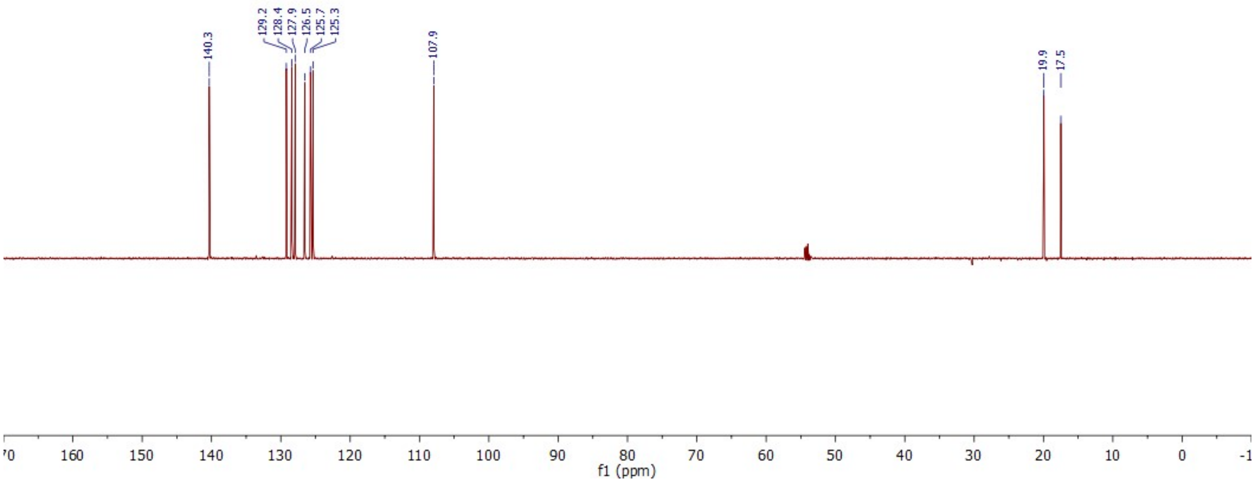
140.3
129.2
128.4
127.9
126.5
125.7
125.3
107.9
19.9
17.5
f1 (ppm)

3. Compound **3:** ^{1}H NMR (500 MHz), ^{13}C and ^{13}Cdept (126 MHz), and ^{11}B NMR (128 MHz) in CD_2Cl_2

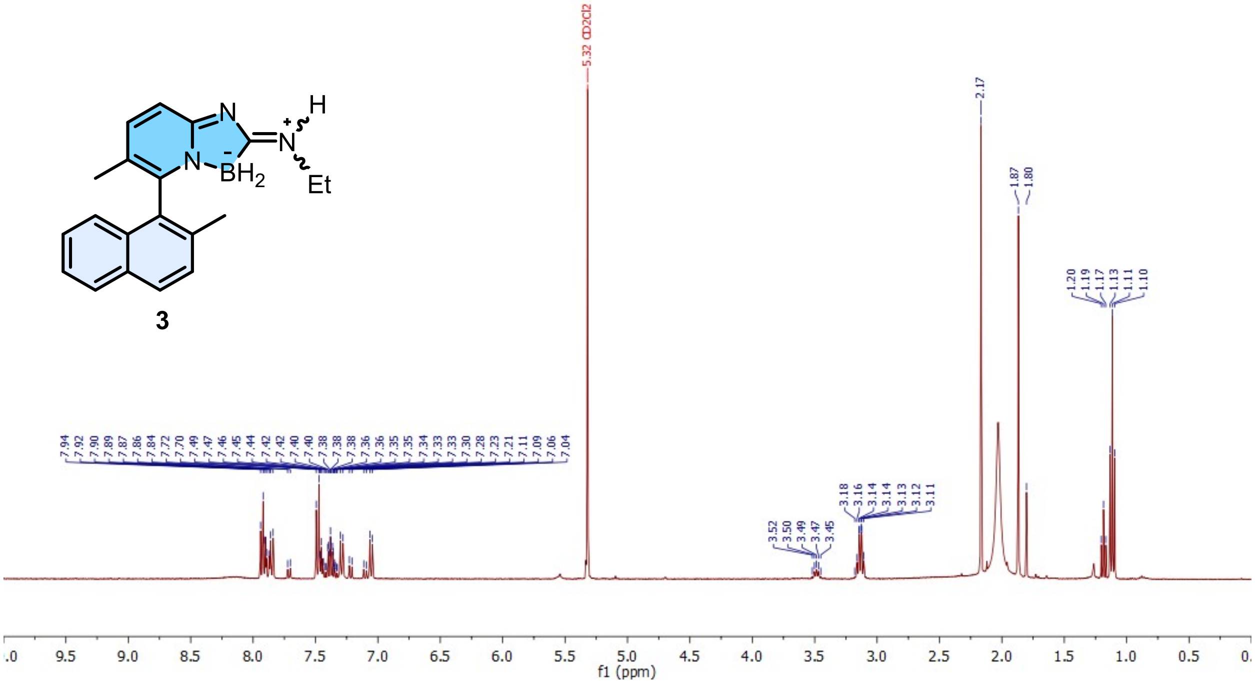

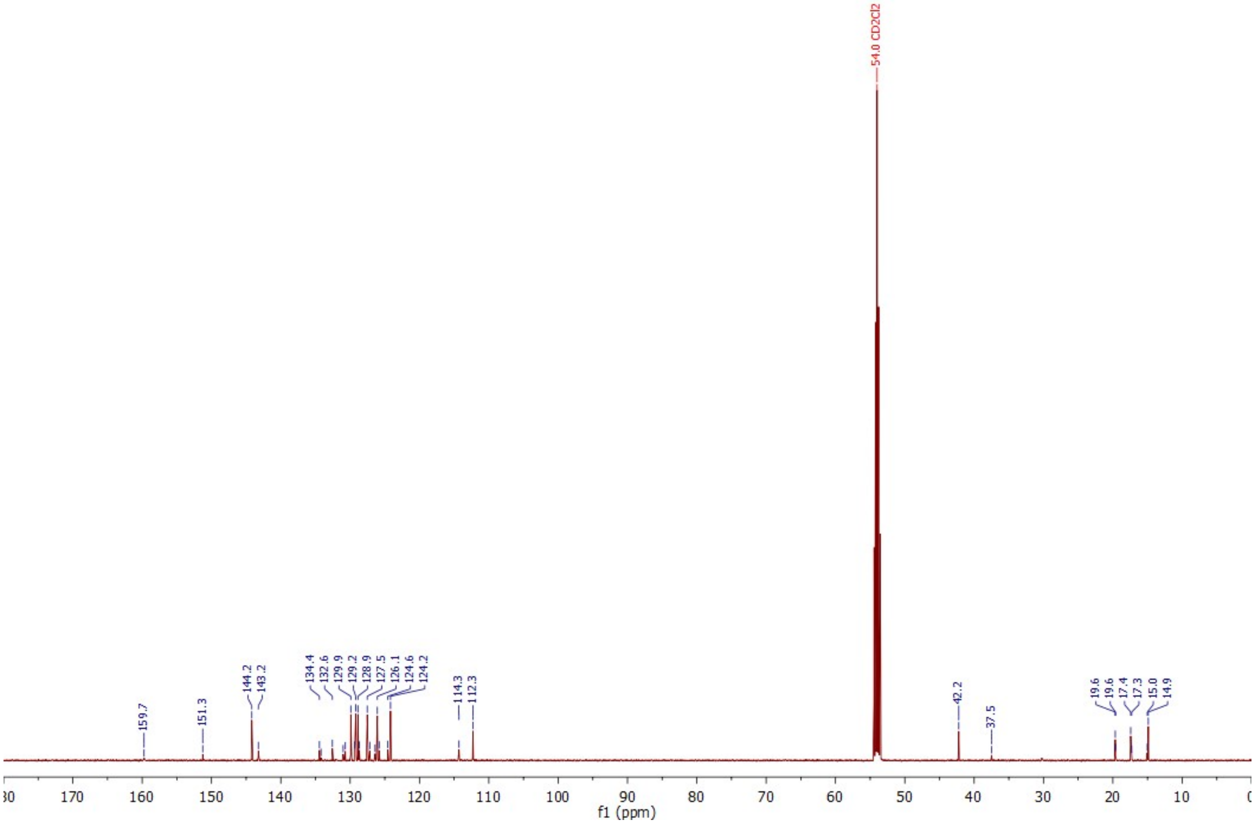

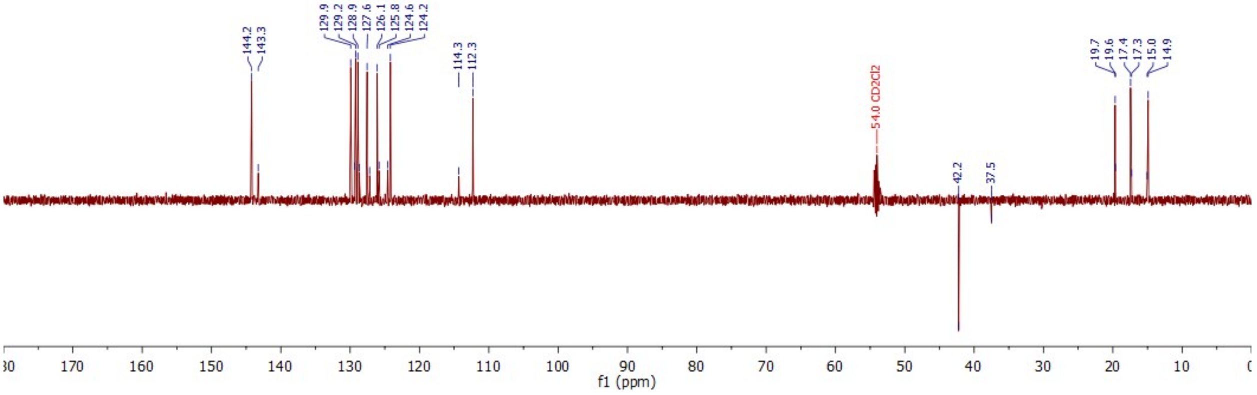
144.2
143.3
129.9
129.2
128.9
127.6
126.1
125.8
124.6
124.2
114.3
112.3
54.0 CD2Cl2
42.2
37.5
19.7
19.6
17.4
17.3
15.0
14.9
f1 (ppm)

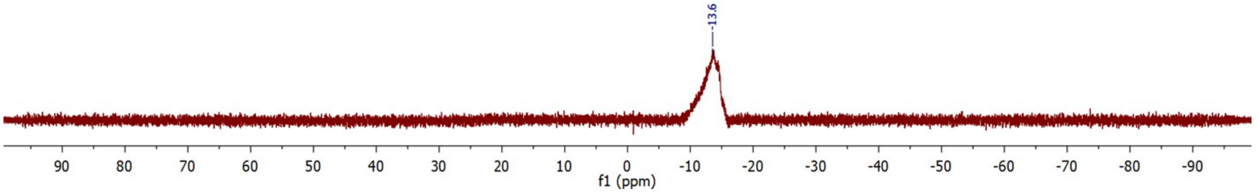
-13.6
f1 (ppm)

X. References

(1) F. Zinna, T. Bruhn, C. A. Guido, J. Ahrens, M. Bröring, L. Di Bari, G. Pescitelli, *Chem. Eur. J.* **2016**, *22*, 16089-16098.

(2) B. Lang, S. Mosquera-Vázquez, D. Lovy, P. Sherin, V. Markovic, E. Vauthey, *Rev. Sci. Instrum.* **2013**, *84*, 073107.

(3) Gaussian 16, Revision A.03, M. J. Frisch, G. W. Trucks, H. B. Schlegel, G. E. Scuseria, M. A. Robb, J. R. Cheeseman, G. Scalmani, V. Barone, G. A. Petersson, H. Nakatsuji, X. Li, M. Caricato, A. V. Marenich, J. Bloino, B. G. Janesko, R. Gomperts, B. Mennucci, H. P. Hratchian, J. V. Ortiz, A. F. Izmaylov, J. L. Sonnenberg, D. Williams-Young, F. Ding, F. Lipparini, F. Egidi, J. Goings, B. Peng, A. Petrone, T. Henderson, D. Ranasinghe, V. G. Zakrzewski, J. Gao, N. Rega, G. Zheng, W. Liang, M. Hada, M. Ehara, K. Toyota, R. Fukuda, J. Hasegawa, M. Ishida, T. Nakajima, Y. Honda, O. Kitao, H. Nakai, T. Vreven, K. Throssell, J. A. Montgomery, Jr., J. E. Peralta, F. Ogliaro, M. J. Bearpark, J. J. Heyd, E. N. Brothers, K. N. Kudin, V. N. Staroverov, T. A. Keith, R. Kobayashi, J. Normand, K. Raghavachari, A. P. Rendell, J. C. Burant, S. S. Iyengar, J. Tomasi, M. Cossi, J. M. Millam, M. Klene, C. Adamo, R. Cammi, J. W. Ochterski, R. L. Martin, K. Morokuma, O. Farkas, J. B. Foresman, and D. J. Fox, Gaussian, Inc., Wallingford CT, 2016.

(4) T. Lu, F. Chen, *J. Comput. Chem.* **2012**, *33*, 580-592.

(5) F. Neese, WIREs Computational Molecular Science 2012, 2, 73-78.

(6) E. van Lenthe, J. G. Snijders, E. J. Baerends, *J. Chem. Phys.* **1996**, *105*, 6505-6516.

(7) D. P. Martin, P. G. Blachly, J. A. McCammon, S. M. Cohen, *J. Med. Chem.* **2014**, *57*, 7126-7135.

(8) Y. Lebedev, C. Apte, S. Cheng, C. Lavigne, A. Lough, A. Aspuru-Guzik, D. S. Seferos, A. K. Yudin, *J. Am. Chem. Soc.* **2020**, *142*, 13544-13549.

(9) CrysalisPro Software system, RIGAKU

(10) G. M. Sheldrick, *Acta Crystallogr., Sect. A: Found. Adv.* **2015**, *71*, 3-8.

(11) G. M. Sheldrick, SHELX-97. *Program for the Analysis of Crystal Structures* **1997**.

(12) O. V. Dolomanov, L. J. Bourhis, R. J. Gildea, J. A. Howard, H. Puschmann, *J. Appl. Crystallogr.* **2009**, *42*, 339-341.
